# Impacts of environmental stress on resistance and resilience of algal‐associated bacterial communities

**DOI:** 10.1002/ece3.8184

**Published:** 2021-10-06

**Authors:** Kathryn Lee Morrissey, Ljiljana Iveša, Soria Delva, Sofie D'Hondt, Anne Willems, Olivier De Clerck

**Affiliations:** ^1^ Phycology Research Group Department of Biology Ghent University Ghent Belgium; ^2^ Center for Marine Research Ruđer Bošković Institute Rovinj Croatia; ^3^ Laboratory of Microbiology Department of Biochemistry and Microbiology Ghent University Ghent Belgium

**Keywords:** climate change, Holobiont, morphological niche, resilience, resistance

## Abstract

Algal‐associated bacteria are fundamental to the ecological success of marine green macroalgae such as *Caulerpa*. The resistance and resilience of algal‐associated microbiota to environmental stress can promote algal health and genetic adaptation to changing environments. The composition of bacterial communities has been shown to be unique to algal morphological niches. Therefore, the level of response to various environmental perturbations may in fact be different for each niche‐specific community. Factorial in situ experiments were set up to investigate the effect of nutrient enrichment and temperature stress on the bacterial communities associated with *Caulerpa cylindracea*. Bacteria were characterized using the 16S rRNA gene, and the community compositions were compared between different parts of the algal thallus (endo‐, epi‐, and rhizomicrobiome). Resistance and resilience were calculated to further understand the changes of microbial composition in response to perturbations. The results of this study provide evidence that nutrient enrichment has a significant influence on the taxonomic and functional structure of the epimicrobiota, with a low community resistance index observed for both. Temperature and nutrient stress had a significant effect on the rhizomicrobiota taxonomic composition, exhibiting the lowest overall resistance to change. The functional performance of the rhizomicrobiota had low resilience to the combination of stressors, indicating potential additive effects. Interestingly, the endomicrobiota had the highest overall resistance, yet the lowest overall resilience to environmental stress. This further contributes to our understanding of algal microbiome dynamics in response to environmental changes.

## Graphical abstract

The resistance and resilience of algal‐associated microbiota to environmental stress can promote algal health and genetic adaptation to changing environments. Bacteria associated with separate morphological niches along the thallus of *Caulerpa cylindracea* differed significantly in response to environmental perturbations, further contributing to our understanding of algal microbiome dynamics.

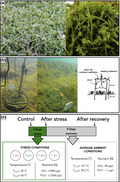

## 1 INTRODUCTION

Eukaryotic organisms across all kingdoms of life are host to complex interactions with microbial partners, otherwise known as their microbiota (Relman, 2008). Associated bacteria, viruses, unicellular eukaryotes (protists), and fungi colonize host surfaces (Wahl et al., 2012), as well as inter‐ and intracellular spaces (Reinhold‐Hurek & Hurek, 2011). Symbiotic relationships with key microbial groups have evolved over time, forming integral functional dependencies, giving rise to the concept of a “holobiont” (Reshef et al., 2006; Rosenberg et al., 2007). While some research has shown associated bacterial species to be host‐specific (Franzenburg et al., 2013; Grünwald et al., 2010; Hollants et al., 2013; Naim et al., 2014), other studies have alluded to the fact that these interactions are interchangeable as long as functional stability is maintained (Burke et al., 2011; Roth‐Schulze et al., 2018). High interindividual variability is often observed for host‐associated microbial communities (Bashan et al., 2016) and the definition of a taxonomic “core” microbiome remains unresolved (Shade & Handelsman, 2012). Alternatively, in some systems a functional core has been alluded to suggest that the microbiota composition is driven by functional requirements related to host growth and adaptation (Burke et al., 2011; Pita et al., 2018; Turnbaugh et al., 2009). Microbes can be recruited from the environment and are either temporarily associated with the host or integrated into the hologenome (Zilber‐Rosenberg & Rosenberg, 2008). This suggests that co‐evolution of the hologenome is continuous and that the microbiota is assembled through a combination of evolutionary and ecological processes (Lemanceau et al., 2017). However, the current theory regarding the hologenome may be inherently flawed by focusing only on host‐driven selection for microbial communities. This has been recently revised to view the host–microbiome as a dynamic ecological community with individual microbial components being influenced by a range of selection pressures (Douglas & Werren, 2016).

The bacterial component of a host–microbiota has been described as highly dynamic with many factors involved in shaping these communities (Rosenberg & Zilber‐Rosenberg, 2018). Both deterministic and stochastic processes have been shown to drive bacterial recruitment, and these processes may vary in effect over time (Zhou et al., 2014). Substantial variability is observed for most host‐associated bacterial communities across geographic regions, under different environments, for different species and even between individuals (Aires et al., 2016; Fraser et al., 2009; Gilbert et al., 2018; Groussin et al., 2017; Hollants, Leliaert, Verbruggen, De Clerck, et al., 2013; Li et al., 2006; Martiny et al., 2006). However, host‐associated bacterial communities have been shown to demonstrate partner fidelity and the potential shifts in these communities are assumed to relate to changing abiotic or biotic conditions (Douglas & Werren, 2016).

Disturbances in environmental conditions such as increased temperature and nutrient load have been observed globally both occurring as long‐term “press” perturbations and short‐term “pulse” perturbations (Bender et al., 1984; Fong & Fong, 2018; Hobday et al., 2016). Increased nutrient load can lead to eutrophication of coastal waters and has been linked to changes in macroalgal community assemblages (Druon et al., 2004; Fong & Fong, 2018). Pulse marine heatwaves, also known as extreme temperature events (Hobday et al., 2016), have been shown to impact coastal benthic algal communities (Duarte et al., 2018; Gouvêa et al., 2017; Wernberg et al., 2016) causing temperature stress that results in shifts in algal‐associated microbes (Campbell et al., 2011; Mensch et al., 2016; Qiu et al., 2019). These extreme temperature events have been predicted to increase in frequency, intensity, and duration as a result of climate change (Perkins et al., 2012). Research has shown that due to the frequent occurrence of extreme temperature events in the Mediterranean sea, coastal populations in this region are highly vulnerable (Christidis et al., 2015, Oliver et al., 2018, Rahmstorf and Coumou, 2011), with one of the highest predicted rises in sea surface temperature to be in the Adriatic sea (Darmaraki et al., 2019).

Changes in environmental conditions impact the overall stability of coastal ecosystems, including macroalgae and their associated bacteria (Egan et al., 2013; He & Silliman, 2019). Bacterial community stability can be defined as a community‐level response to an environmental disturbance, and incorporates both resistance and resilience, which can be quantified using community metrics (Shade et al., 2012). The resistance of a community is the extent to which the community structure remains stable in response to a perturbation, whereas resilience is defined as the rate at which the community reverts back to its original state. Microbiome stability is either indicated by taxonomic compositional structure or functional capability that is resistant to environmental perturbation, having the ability to return to the previous stable state (Allison & Martiny, 2008; Coyte et al., 2015). Bacterial community stability can be enhanced through the phenotypical plasticity of key microbes (Shade et al., 2012) as well as the functional redundancy observed for many bacterial groups (Bashan et al., 2016; Burke et al., 2011).

In the marine environment, extensive microbiome research has been done on sessile organisms such as sponges, corals, and macroalgae (Egan et al., 2013; Rosenberg et al., 2007; Webster & Thomas, 2016). Marine macroalgae, commonly referred to as seaweeds, are known to harbor highly diverse bacterial communities that demonstrate a niche specificity corresponding to the microscale location either within the endosphere, as part of the epi‐biofilm, or associated with differentiated structures such as holdfasts and rhizoids (Morrissey et al., 2019; Serebryakova et al., 2018). Studies indicate that tight associations between algae and intracellular bacteria represent a form of bacterial inheritance, in which the origin of an algal population can be identified. This has been demonstrated by tracing the origin of the invasive seaweed *Caulerpa taxifolia* in the Mediterranean (Arnaud‐Haond et al., 2017; Burr & West, 1970; Meusnier et al., 2001). Endobionts have been previously demonstrated to be stable over time (Hollants et al., 2013; Meusnier et al., 2001), whereas epibacterial communities have been assumed to be more dynamic as they display temporal changes (Bengtsson et al., 2010; Mancuso et al., 2016). However, growing research suggests that both endo‐ and epibacterial communities are influenced by environmental factors (Aires et al., 2016; Hollants, Leliaert, Verbruggen, Willems, et al., 2013).

There is limited knowledge regarding the underlying principles of microbial assembly and structure (Burke et al., 2011), with even less known about the environmental effects on algal‐associated bacterial communities cross‐differentiated algal structures, also known as morphological niches (Morrissey et al., 2019). Within the marine environment, microbial research has been done on planktonic bacterial communities and communities associated with corals and sponges (Glasl et al., 2018; Lima‐Mendez et al., 2018; Webster & Reusch, 2017; Ziegler et al., 2017). In the field of algal microbiomes, studies have mainly focused on characterizing associated bacteria from natural habitats that differ in environmental parameters and not from in situ simulated experiments (Aires et al., 2016; Bengtsson et al., 2010; Burke et al., 2011; Campbell et al., 2015; Egan et al., 2013; Hollants, Leliaert, Verbruggen, Willems, et al., 2013; Mancuso et al., 2016). Further investigations into the influence of environmental factors on the algal microbiome in situ are necessary to fully understand these complex host–microbiome interactions and the impact this has on host health and function (Egan et al., 2013).

Furthermore, there has been little research to date that investigates algal bacterial community resistance and resilience of different morphological niches in response to environmental perturbations. As bacterial communities have been shown to differ between morphological niches of the same individual (Morrissey et al., 2019; Paix et al., 2020), we therefore hypothesize that bacteria associated with each morphological niche have different community resistance and resilience to stress, and this may have varying implications for total algal microbiome stability. Hence, this study aims to effectively assess these ecological dynamics in situ.

We performed in situ heatwave manipulations and nutrient enrichments within semiclosed mesocosm systems. The aim of these investigations was to characterize the effect of pulse abiotic disturbance on the bacterial communities associated with individual morphological niches of the green algae *Caulerpa cylindracea*, as well as the surrounding environment. We took the naturally occurring, prestress bacterial communities as a baseline and then analyzed the effects of a 3‐day stress duration and a 9‐day recovery period. We assessed the bacterial community changes and calculated microbial resistance and resilience to individual abiotic stressors as well as a combination of the two. In this study, we hypothesize that (a) bacterial community resistance and resilience to environmental perturbation are dependent on morphological niche association, and (b) bacterial community responses differ between types of stress and the combination of two stressors.

## 2 MATERIALS AND METHODS

### 2.1 Sampling and mesocosm design

**FIGURE 1:**
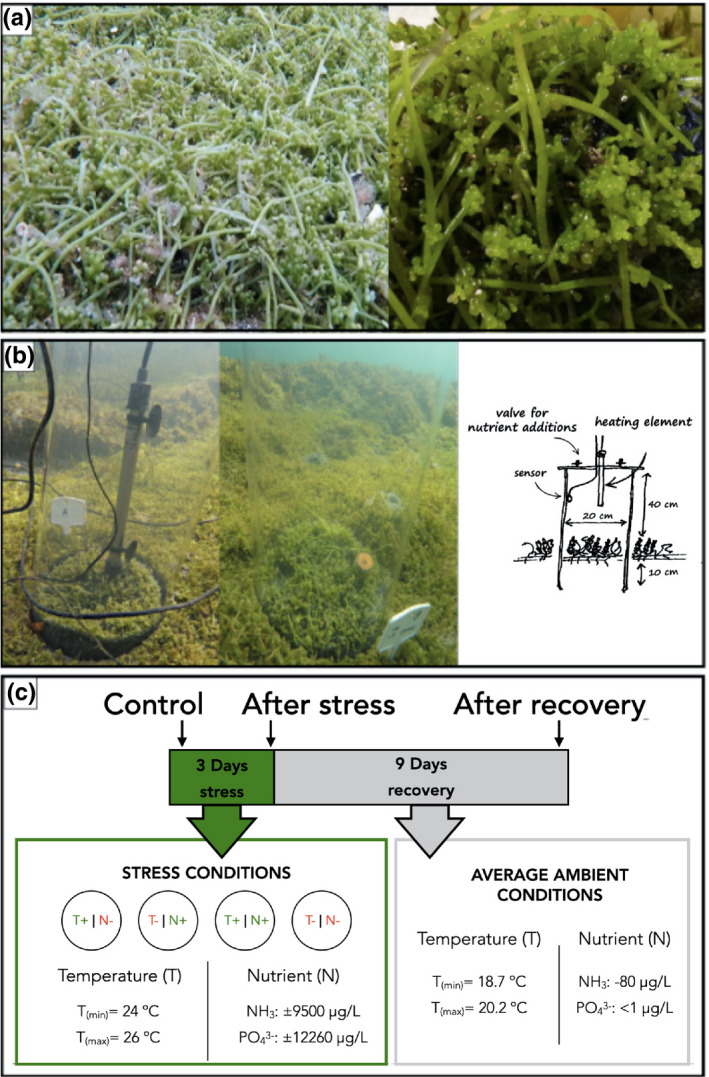
Overview of the site and experimental design of the mesocosms. At the site, Caulerpa cylindracea formed a dense mat on the seafloor (a). Semiclosed mesocosms were designed for each treatment type with and without an integrated heating element (b). Samples were stressed for 3 days, with a recovery period of 9 days and samples were taken at three timepoints, before stress, directly after stress and after recovery (c)

Experiments were conducted situ along the west coast of Istria (northern Adriatic Sea, Croatia; 45.177953°N, 13.593907°E) where *Caulerpa cylindracea* formed a thick mat on shallow subtidal (2–4 m) sand and rock bottoms with frond lengths ranging from 2 to 5 cm (Figure 1a). A total of 12 mesocosm experiments were performed where mesocosm tubes, encompassing a volume of 12.5 L each, were placed randomly at least 2 m apart and assigned a treatment (Figure 1b). Four treatments were carried out: temperature stress, nutrient enrichment, combination of temperature and nutrient, and a control. The treatments were executed for 3 consecutive days. For the temperature manipulations, we simulated pulse heatwave conditions based on the definition used by Sorte et al. (2010), adapted from Meehl and Tebaldi (2004), in which daily maximum sea surface temperatures exceed 3–5°C above normal for a minimum of 3 days. The yearly sea surface temperatures for the region range from approximately 8–27°C (Iveša et al., 2015). For the respective sampling period (October 2016), historical monthly averages (2001–2015) ranged between 16 and 20°C, and ambient temperatures for the experimental days were measured between 17.9 and 21.8°C (Table S1). In our experiment, the mesocosms were actively heated using an imbedded heating element at a rate of ~0.7°C/h, reaching a maximum of ~4.2°C above daily average after 6 h. The maximum temperature was sustained for an additional 6 h, totaling 12 h of active heat treatment. At night (12 h), the mesocosms were not actively heated and therefore allowed to cool down to ambient temperatures. This was repeated for a duration of 3 days. Nutrient enrichment was added in liquid form made from dissolved Compo© universal fertilizer in sterile seawater at a final concentration of approximately 9,550 µg/L nitrogen (supplied as ammonium nitrate), 50 times the maximum recorded natural concentration of dissolved inorganic nitrogen (approximately 191,47 µg/L DIN) previously observed in the region (Djakovac et al., 2012). As a by‐product of the fertilizer, phosphorous (in the form of rock phosphate) was added to the concentration of approximately 12 260 µg/L, well above the natural recorded values of phosphate at approximately 4 µg/L. This was done to simulate a pulse hypereutrophic event (Yang et al., 2008), as the Adriatic Sea has been characterized by the eutrophication risk index (EUTRISK) as a high‐risk area (Druon et al., 2004). After the 3‐day stress period, the mesocosm tubes were removed and sites marked for resampling. The algae were then left to recover for a period of 9 days (Figure 1c). This entire mesocosm setup was repeated three times on separate algal patches selected at random more than 2 m apart. The total duration of the experimental period lasted no longer than 14 days (11 October–25 October 2016). Samples were taken prior to the disturbance, directly after the 3‐day disturbance and after the recovery period. Sampling units (SU) included interconnected thalli (uprights, stolon, and rhizoids). These were further separated in the laboratory into three distinct morphological niches from the same individual, namely, endobiome, epibiome, and rhizobiome fractions and washed with artificial sterile seawater (ASW) at 35 ‰. Epibionts were retrieved by swabbing the surface with a sterile swab and transferring directly into sterile Eppendorf tube. Thalli were surface sterilized following the protocol from Hollants et al. (2010), in which *Caulerpa* thalli were incubated in CTAB buffer with 20 mg/ml proteinase K, and then washed with sterile ASW and incubated overnight in a 1:1 mixture of 0.2‐µm filtered Umonium Master and sterile ASW. Following this, thalli were washed ten times in sterile ASW. To acquire the associated endobionts, rhizoids were separated with a sterile blade after being removed from any attached substrates. Sediment and water samples were also taken for all timepoints and treatments. Approximately 2 g of surface layer sediment surrounding the sampled thalli was placed directly into sterile bags. Water was sampled directly from the environment before stress, directly after stress and after the recovery period. For the individual mesocosms, water was extracted from the mesocosm via syringe through a valve directly after the stress period. For each treatment, 100 ml of water was filtered in triplicate through a 0.2‐μm polycarbonate filter and the filters were then used for downstream analysis. The samples were frozen at −20°C and kept for DNA extraction. The environmental conditions during the full length of the experiment were analyzed (Table S2). Nutrient levels and temperature were measured for the surrounding environment before stress as well as after the recovery period. Mesocosm nutrient levels were also measured directly after in situ manipulations for each treatment.

### 2.2 DNA extraction and 16S metabarcoding

Bacterial DNA was extracted from all samples following the protocol by Doyle and Doyle (1987), with slight modifications (Morrissey et al., 2019). Algal samples were homogenized in liquid nitrogen prior to an additional bead‐beating before adding the CTAB extraction buffer. The 16S rRNA gene amplification was done using the universal primers 27F (5′‐AGAGTTTGATCMTGGCTCAG‐3′) and 1492R (5′‐TACGGYTACCTTGTTACGACTT‐3′) (Lane, 1991). Following a post‐PCR clean‐up with AMPure^®^ bead purification (Beckman Coulter, Inc., CA), a second nested PCR was done to amplify the V5‐V7 hypervariable region of the bacterial 16S rRNA gene using the primers 799mod3F (5′‐GGATTAGATACCKGG‐3′) and 1193R (5′‐ACGTCATCCCCACCTTCC‐3′) to reduce chloroplast contamination (Aires et al., 2012; Bodenhausen et al., 2013; Hanshew et al., 2013). The last round of PCR, the primers included Illumina adaptors used for indexing as part of the library preparation. All PCR amplifications were run with negative controls. The settings of the touchdown PCR were as follows: 5 min at 95°C, followed by 10 touchdown cycles of 1 min at 95°C, 1 min at 65°C, and 3 min at 72°C. The annealing temperature started at 65°C and was reduced to 60°C in increments of 0.5°C per cycle. Upon reaching a minimum annealing temperature of 60°C, another 15 cycles were performed, consisting of 1 min at 95°C, 1 min at 60°C, and 3 min at 72°C. A final elongation step was performed for 20 min at 72°C. Following PCR analysis, amplicons were purified using AMPure^®^ bead purification and subsequently examined for successful amplification by agarose gel electrophoresis. An index PCR was performed to add Illumina sequencing adapters and dual indices to the end of each amplicon using the Nextera XT Index Kit (Illumina, San Diego, USA). Indexed amplicons were purified using AMPure beads, and DNA concentrations were quantified using the Qubit dsDNA Broad Range Assay Kit (Invitrogen, Carlsbad, USA). Quantified PCR products were then pooled at equimolar concentration and sent to BaseClear B.V. for Illumina MiSeq v.3 (2 × 300 bp) sequencing.

### 2.3 Bacterial community analysis and characterization

Quality assessment of the retrieved sequences was performed using fastqc. Primers were then removed, and reads shorter than 260 bp were removed. Read pairs were then merged using the BBMerge function as a part of the BBTools package (Bushnell et al., 2017). Merging was done with a minimum overlap of 100 bp, and no gaps were allowed in the overlapping region of the aligned reads. Additional quality filtering was done by setting the maximum expected error at 0.5, and assembled reads longer than 420 bp were discarded. After preprocessing, sequences were processed using the UPARSE pipeline (Edgar, 2013) within the USEARCH sequence analysis package (Edgar, 2010). Unique sequences were identified and OTUs were clustered at 97% similarity based on the UPARSE‐OTU algorithm, which simultaneously removes chimeric sequences and singletons were removed. Taxonomy was assigned to the genus level at a confidence of 90% using the RDP database release 11 (Cole et al., 2014) in Mothur v. 1.36.1 (Schloss et al., 2009). In qiime 2 (Caporaso et al., 2010), the OTU output table, metadata file, reference sequences, and phylogenetic tree, constructed using FastTree2 (Price et al., 2010), were merged into a biom file with JSON format. All downstream statistical analyses were done using R software 3.5.2 (R Development Core Team, 2010).

### 2.4 Data analysis

Data import and preprocessing were done using the “phyloseq” package (McMurdie & Holmes, 2013) and “microbiome” R‐package (Lahti et al., 2017). Cyanobacterial sequences as well as potential nonprokaryote contamination sequences, such as chloroplasts and mitochondria, were removed (Mancuso et al., 2016) resulting in 8,563 taxa and total 5,019,077 reads. Samples were rarified to 2,821 reads per sample and normalized using the compositional transformation (Lahti et al., 2017). Multivariate analysis of variance tests with permutations (PERMANOVA) was done on the complete data set using the *adonis2* function included in the “vegan” R‐package version 2.4‐6 (Oksanen et al., 2016). The PERMANOVA tested the effect of morphological niche, sequencing run, experiment number, timepoint, treatment, and replicate number, using Bray–Curtis dissimilarity. Beta‐diversity of the bacterial communities was explored for each sample type, and a principle coordinates analysis (PCoA) ordination plot was generated to visualize the bacterial community variation observed. For only the algal samples, Bray–Curtis dissimilarities were calculated for the bacterial communities in comparison with the control treatments for each morphological niche. These distances were then plotted for each treatment. To analyze the effect of each treatment on the community similarity after the stress and recovery periods, we performed a one‐way analysis of variance (ANOVA) and calculated the least‐squares means with the *lsmean*s function in “emmeans” R‐package version 2.30‐0 (Lenth, 2016) to compute the contrasts between treatments. We then determined the significance of the effects with general linear hypotheses in combination with the single‐step method of multiple testing correction using “multcomp” R‐package version 1.4‐10 (Hothorn et al., 2008). The individual taxa significantly different in abundance and occurrence between treatments and timepoints were investigated using the *multipatt* function in the “indicspecies” R‐package using the association index “IndVal.g” (De Cáceres & Legendre, 2009; De Cáceres et al., 2010; Dufrene & Legendre, 1997).

### 2.5 Functional prediction

Functional changes in response to environmental stress were inferred by assigning functional attributes to the OTU identifications of the 16S rRNA gene using the Phylogenetic Investigation of Communities by Reconstruction of Unobserved States (PICRUSt2) (Douglas et al., 2019). Functional predictions were performed on the entire raw dataset, and KEGG orthologies (KOs) were assigned to each OTU with a NSTI cutoff of <2 as recommended by the authors. The data were then corrected by 16S copy number to generate a prediction of the full functional profile of the dataset. Data were rarified to account for uneven depth. Functional similarities of the bacterial communities associated with each morphological niche were compared to the control for each treatment directly after stress (3 days) and after the recovery period (12 days). This was done using a one‐way analysis of variance (ANOVA) on the Bray–Curtis dissimilarities of treatment samples compared to the control. Significance of each treatment was determined by general linear hypotheses using “multcomp” (R‐package version 1.4‐10)(Hothorn et al., 2008) based on *lsmean*s function in “emmeans” (R‐package version 2.30‐0) and corrected for multiple testing (Lenth, 2016). KOs contributing to the observed differences in community functions were identified using the *multipatt* function in the “indicspecies” R‐package (De Cáceres & Legendre, 2009; De Cáceres et al., 2010; Dufrene & Legendre, 1997).

### 2.6 Calculating community resistance and resilience

Bacterial communities were compared on both an OTU level and a functional level using Bray–Curtis dissimilarity. This value was used as a community metric to represent community dissimilarity between samples taking into account both composition and abundance. Distances were calculated from the control community sampled at each timepoint. Controls across all three timepoints were tested against each other to determine temporal variability. Using the community distances as a response variable, indexes of the resistance and resilience of the microbial communities were calculated using equations defined by Orwin and Wardle (2004).

The resistance index was calculated as:

$$RS=1‐\frac{2\left|C_{1}‐D_{1}\right|}{C_{1}+\left|C_{1}‐D_{1}\right|}$$

where C₁ represents the control bacterial community dissimilarity directly after stress, and D₁ represents the bacterial community for the respective treatment directly after stress.

The resilience index was calculated as:

$$RL=\frac{2\left|C_{1}‐D_{1}\right|}{\left|C_{1}‐D_{1}\right|+\left|C_{2}‐D_{2}\right|}‐1$$

where C₂ represents the control bacterial community dissimilarity after the recovery period and D₂ represents the bacterial community for the respective treatment after the recovery period.

## 3 RESULTS

### 3.1 Bacterial community composition

**FIGURE 2:**
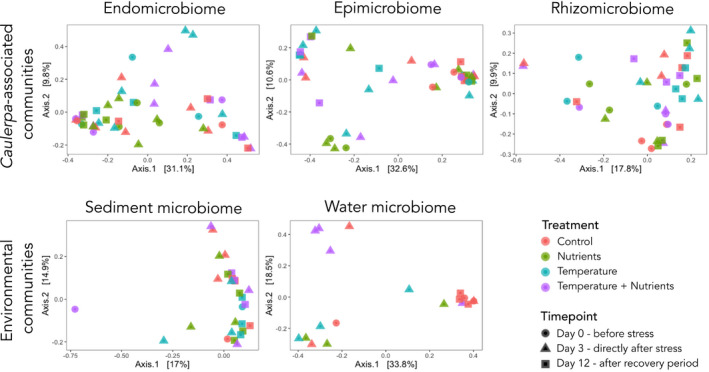
PCoA plots of the Bray–Curtis Dissimilarity between bacterial communities associated with each sample type for each treatment (pink—control; green—nutrients; blue—temperature; purple—combination treatment) and timepoint (circle—day 0; triangle—day 3; square—day 12)

A total of 12,244,523 raw paired‐end reads of the V5–V7 region of the 16S rRNA gene were obtained from two sequencing runs (MiSeq v3 platform) and after filtering 5,118,598 high‐quality merge pairs remained. Sequencing run was added as a covariate to our analyses and did not significantly contribute to any differences observed (*R* ^2^ < .005; *p* = .184). Sequences were binned into a total of 8,563 OTUs at a 97% similarity level, with an average of 28,037 (± 18,678) reads per sample. 2,198 OTUs were removed via rarefaction (Figure S1). The number of OTUs varied across sample types, with only 209 OTUs shared between morphological niches out of the total 2,718 OTUs present in the bacterial communities for all timepoints (Figure S2). The beta‐diversity of the bacterial communities was assessed and compared visually using a principal coordination analysis ordination plot (Figure S3). The bacterial communities clustered according to their associated niche, with the rhizosphere and sediment visually showing the most overlap. The epimicrobiome samples showed the widest spread, overlapping with the endomicrobiome, rhizomicrobiome, and the bacteria in the water column. Further investigations into the ordination plots of the separate timepoints of each treatment for each individual morphological niche indicated that there were no clear visual separations based on the timepoints of each treatment (Figure 2). The taxonomic structure (Figure S4) of the algal samples showed differences between morphological niches. Variability in taxonomic composition was observed between samples of the same morphological niche, both over temporal scales of the controls as well as between treatments (Figure S3). However, the most abundant bacterial class consistently found across all samples of the endo‐ and epimicrobiome was Gammaproteobacteria, with Deltaproteobacteria most abundant in the rhizomicrobiome.

### 3.2 Changes to algal‐associated bacteria in response to environmental stressors

**FIGURE 3:**
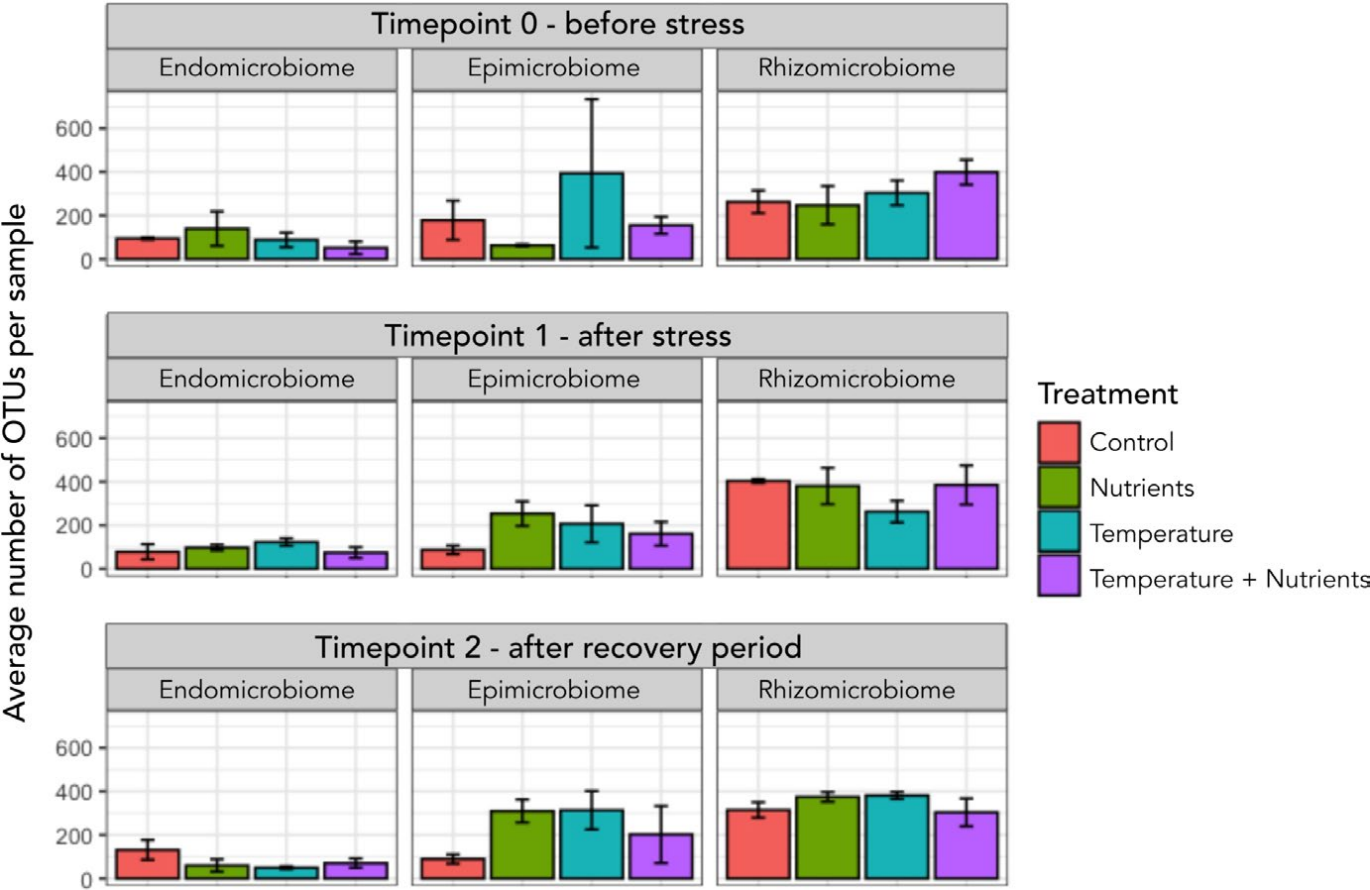
Mean OTU numbers ± SE for each morphological niche at each timepoint for each treatment

For the algal samples, the number of OTUs was significantly different between morphological niches (Figure 3, Table S3). The epimicrobiome had the highest OTU richness at 3,254, whereas the rhizomicrobiome had 2,945 and the endomicrobiome had 1,499. The number of OTUs observed under different treatments revealed the epimicrobiome to be more variable over treatments and timepoints; however, this was not statistically significant (Figure 3, Table S3). Overall, the rhizomicrobiome showed to have significantly more OTUs in each sample (Figure 3, Table S3), as well as more OTUs uniquely assigned to the rhizomicrobiome (Figure S2).

**FIGURE 4:**
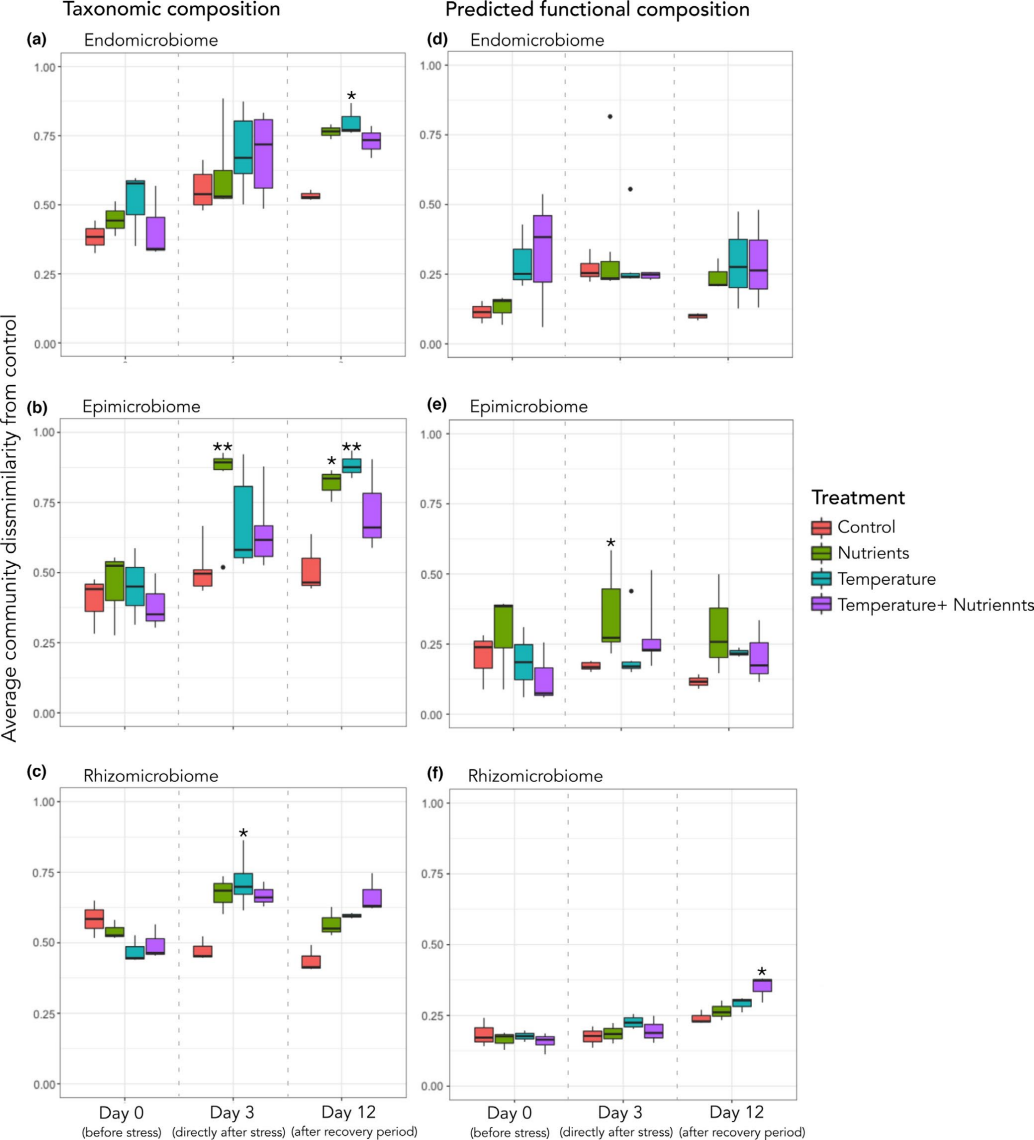
Community dissimilarity (Bray–Curtis) for both taxonomic composition (a‐c) and predicted functional profiles (d‐f) over time for each algal morphological niche. (a) Endomicrobiome taxonomy, (b) endomicrobiome predicted functions, (c) epimicrobiome taxonomy, (d) epimicrobiome predicted functions, (e) rhizomicrobiome taxonomy, (f) rhizomicrobiome predicted functions. (Significant treatments are indicated by *, relating to Tables S5 and S7)

Comparing the overall bacterial community similarities indicated that morphological niche showed a significant influence on the bacterial communities (Table S4). Investigating this further by univariate tests for the effect of the treatments on each algal morphological niche at each timepoint, we observed that the control did not indicate significant differences (Table S5). The effects of the treatments on bacteria associated with each morphological niche are tabulated in Table S5. Treatment effects on the endomicrobiome did not show any significant difference on the community composition directly after stress. However, the effect of temperature showed a significant difference at timepoint 2, after the recovery period of 9 days (Figure 4, Table S5). The epimicrobiome experienced a significant change (glht, Tukey's post hoc test, estimate = 0.327; *SE* = 0.075; *p* = .001) immediately after nutrient enrichment and continued to be significantly different after the recovery period (glht, Tukey's post hoc test, estimate = 0.301; *SE* = 0.1; *p* = .047) (Figure 4, Table S5). Heat stress seemed to have a delayed effect on the epibacterial community with a significant difference observed only after the recovery period (glht, Tukey's post hoc test, estimate = 0.371; *SE* = 0.1; *p* = .008). Each stressor, as well as the combination, had an effect on the rhizomicrobiome bacterial communities after stress (glht, Tukey's post hoc test, Nutrients: estimate = 0.2, *SE* = 0.049, *p* = .003; Temperature: estimate = 0.245, *SE* = 0.046, *p* < .001; Temp + Nutr: estimate =0.195, *SE* = 0.049, *p* < .005); however, the bacterial community managed to recover from the effects of the nutrient stress (glht, Tukey's post hoc test, estimate = 0.109; *SE* = 0.044; *p* = .154).

The species indicator analysis (De Cáceres & Legendre, 2009; De Cáceres et al., 2010; Dufrene & Legendre, 1997) revealed a total of 104 OTUs with a significant difference in occurrence and or abundance between treatments and timepoints (Table S6). Of these, 42 OTUs remained unclassified at the family level, 31 at the order level, 20 at the class level, and only 5 at the phylum level. Of the species classified to family level, the species that associated with the temperature and combination treatments for all the morphological niches and timepoints belonged predominantly to the Rhodobacteraceae family. Those that associated exclusively to the nutrient treatments belonged to the Desulfobulbaceae, Sphingomonadaceae, Enterobacteriaceae, Xanthomonadaceae, and Iamiaceae families. When considering the bacterial communities that had a significant difference from the control (Table S5), 5 indicator species were exclusively associated with the endomicrobiome directly after temperature stress, 4 of which were associated with the Rhodobacteraceae family and one to an unclassified Oceanospirillales. Only one indicator species was identified exclusively for the epimicrobiome directly after nutrient stress, belonging to the Enterobacteriaceae family, whereas no significant indicator species were exclusively associated with the differences observed for the epimicrobiome after the recovery period. Species belonging to the Rhodobacteraceae, Sphingomonadaceae, and Desulfobulbaceae families, as well as an unclassified Cytophagales, unclassified Clostridiales, unclassified Chromatiales, and several unclassified Gammaproteobacteria, contribute to the differences in community structure observed for the temperature stress treatment for the epimicrobiome after the recovery period. Lastly, the significant difference observed of the rhizomicrobiome directly after the temperature stress is associated with 5 species assigned to unclassified classes of Bacteroidetes, and Desulfobacteraceae and Desulfobulbaceae families (Table S6).

### 3.3 Predicted functional responses of bacterial communities to environmental stressors

Inferred functional annotations were analyzed using PICRUSt2 (Douglas et al., 2019), in which each OTU was assigned to at least one or more KO. Some OTUs were assigned to more than one function leading to 7,191 functional representatives. The predicted functional profiles of each morphological niche for each treatment showed no significance between controls (Table S7). A significant difference in community functional profile was observed for the epimicrobiome directly after the nutrient stress (glht, Tukey's post hoc test, estimate = 0.189; *SE* = 0.061; *p* = .039), but not after the recovery period. In the rhizomicrobiome, functional profiles were not significantly different, except for after the recovery period for the combination stress samples (glht, Tukey's post hoc test, estimate = 0.092; *SE* = 0.03; *p* = .042). None of the treatments had any significant effect on the functional capacities of the endomicrobiome (Table S7). A full list of the individual functions significantly contributing to variation observed between samples at timepoint 1 (directly after stress) and timepoint 2 (after the recovery period) is found in Table S7. Directly after stress, 58, 58, and 29 significantly different functions were identified for the endomicrobiome, epimicrobiome, and rhizomicrobiome, respectively. After the recovery period, the number of significantly different functions identified for the endomicrobiome, epimicrobiome and rhizomicrobiome were 65, 115, and 75, respectively. Many functions were associated as indicators for more than one treatment. Significant functions solely associated with the epimicrobiome directly after nutrient stress were related to EPS production (succinoglycan biosynthesis proteins ExoW and ExoO; endo‐1,2‐1,4‐beta‐glycanase ExoK), several metabolic reactions (glucuronokinase; glycogen synthase; maleylacetate reductase; propionate kinase), and RNA synthesis (DNA‐directed DNA polymerase subunit beta‐beta). Functions potentially contributing to the observed functional differences in the rhizomicrobiome exposed to the combination stress after the recovery period were related to membrane transport (MFS transporters, D‐allose transport system permease protein, bicarbonate transport system ATP‐binding protein), modifications to the 23S rRNA component (23S rRNA (adenine‐N6)‐dimethyltransferase), and the synthesis of sphingolipids (neutral ceramidase) (Table S8).

### 3.4 Resistance and resilience of bacterial communities

**FIGURE 5:**
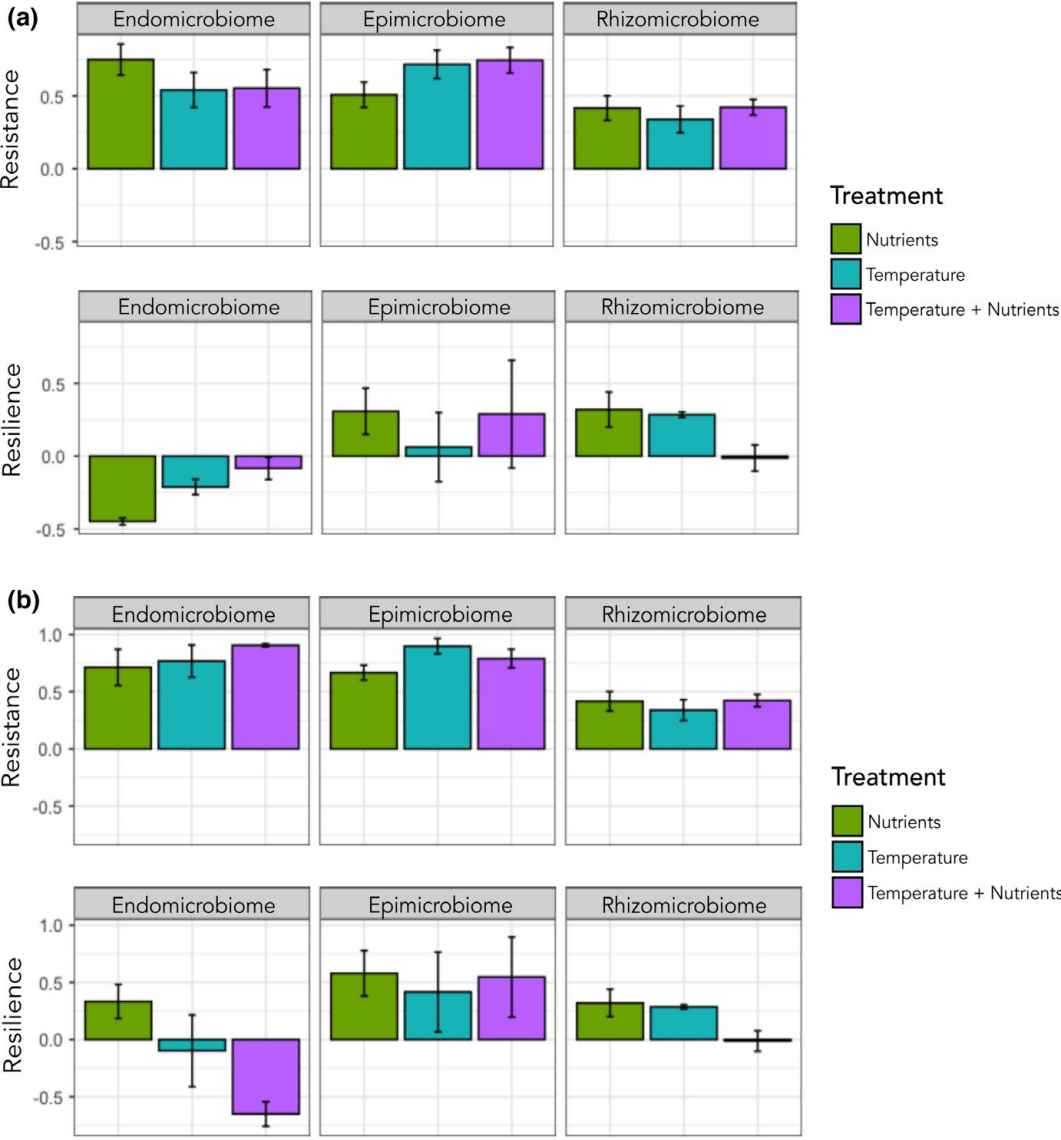
The taxonomic (a) and the predicted functional (b) resistance and resilience index calculated for each morphological niche in response to different treatment types, using Bray–Curtis dissimilarity values as a community metric. Resistance index shown as a value between 1 and 0, with 1 indicating complete resistance and 0 no resistance. Resilience index shown as a value between 1 and −1, with positive values indicating recovery to control state, near‐zero values indicating no change between poststress state and postrecovery period state, and negative values indicating a state of ongoing change

The resistance index is shown as value between 1 and 0 for each bacterial community, with 1 indicating complete resistance and 0 representative of no resistance to the perturbation. The resilience is displayed as an index value between 1 and −1, with positive values representing that the bacterial communities post recovery are potentially recovering to the control state. Resilience values near zero indicate that the bacterial communities post recovery period are at the same state as those post stress, and negative resilience index values indicate that these communities that are still undergoing changes. Taxonomic and functional resistance and resilience were assessed for each morphological niche under different treatments (Figure 5a). It is observed that the endomicrobiome taxonomic composition is fairly resistant to the influences of the treatments, with none of the treatments showing a drop below 0.5. The highest resistance in the endomicrobiome taxonomic composition is seen for the nutrient enrichment. In contrast, the epimicrobiome taxonomic composition under increased nutrient load showed the lowest resistance for that morphological niche, indicating that the microbial taxonomic structure is less resistant to nutrient stress than the other treatments. For the microbial resilience, the endomicrobiome exhibits the least taxonomic resilience overall, with the separate nutrient and temperature treatments indicating the communities are still experiencing shifts in the taxonomic structure of the community, either temporal or treatment related or both. The epi‐ and rhizomicrobiome show higher taxonomic resilience than the endomicrobiome with index values between 0.5 and 0. For both, the epi‐ and rhizomicrobiome communities under nutrient stress had the highest resilience index. The resilience index for the temperature treatment was near zero for the epimicrobiome, whereas the rhizomicrobiome had a resilience index of approximately zero for the combination treatment. The functional resistance and resilience of the epimicrobiome and rhizomicrobiome follow the same patterns as the taxonomic resistance and resilience previously mentioned (Figure 5b), barring the increased functional resilience of the epimicrobiome to the temperature stress. The functional composition of the endomicrobiome also exhibits a high resistance to stress as is observed with the taxonomic resistance. However, differences are observed in the functional resilience when compared to the taxonomic resilience of the endomicrobiome. Functional composition is more resilient to nutrient stress, and the resilience index for the combination treatment indicates the functional profiles are still fluctuating.

## 4 DISCUSSION

This study documents the taxonomic and predicted functional changes in bacterial communities associated with the green alga *Caulerpa cylindracea* in response to in situ pulse disturbances of environmental conditions. Resistance was calculated as the immediate changes in bacterial community structure after a period of abiotic stress, and resilience was determined as the potential recovery of the bacterial community to the original state before disruption. Natural variation in the community composition of marine bacteria has been observed, with a large proportion attributed to unexplained variables or stochasticity (Hollants, Leliaert, Verbruggen, De Clerck, et al., 2013; Lima‐Mendez et al., 2018). The results in this study support this notion as a high degree of variability in bacterial communities was observed between replicates (Figure 2). However, environmental parameters, macroalgal hosts species, grazers, pH, and surface chemical metabolites have been shown to influence bacterial composition (Aires et al., 2016; Egan et al., 2014; Li et al., 2006; Martiny et al., 2006; Saha & Weinberger, 2019; Wahl et al., 2012). Furthermore, distinct microbial communities have been shown to associate with specific niches of algal thalli dependent on the specific functional requirements of the differentiated tissue or pseudo‐tissue type (Morrissey et al., 2019; Paix et al., 2020; Serebryakova et al., 2018). Our results show that bacteria associated with specific algal morphological niches have unique responses to different stressors.

Research has suggested that endobionts are more stable over time (Hollants, Leliaert, Verbruggen, Willems, et al., 2013; Meusnier et al., 2001) than epibionts, which are naturally more exposed and therefore more susceptible to changing environments (Bengtsson et al., 2010; Mancuso et al., 2016). The thought extends further into the nature of these bacteria with the underlying assumption that endobionts are more tightly associated with their respective host with symbioses co‐evolved over time (Aires et al., 2013; Arnaud‐Haond et al., 2017). In this study, endobionts were observed to have a higher taxonomic and functional resistance overall in comparison with the epi‐ and rhizomicrobiome. However, the resilience of both the taxonomic structure and predicted functional profiles was low for the endomicrobiome under all types of stress, although the community differences for the nutrient and combination stress were not significantly different from the control after the recovery period. As the resilience metric takes into account the disparity between community differences over time, a low initial difference directly after the stress compared to a greater difference observed after the recovery period would result in a lower resilience metric, even though it is not statistically significant. Therefore, the resilience index can be helpful in highlighting communities still undergoing taxonomic and functional shifts as a response to perturbation. Our results show that for the endomicrobiome, taxonomic composition resilience is negative, while the functional resilience is positive for the nutrient stress. This suggests that although the community structure is still changing, it is recovering functional capacity. Alternatively, for the temperature and combination treatments, both the endomicrobiome taxonomic and functional resilience indexes indicate they have either shifted to a new state or are still fluctuating, therefore, suggesting that the temperature treatment and the combination of treatments have a larger impact of endomicrobial communities. The resistance profiles for the epimicrobiome showed the highest sensitivity to the nutrient treatments for both taxonomic and functional composition. The functional profiles of the individual treatments showed a higher resilience than the taxonomic structure for the epimicrobiota, suggesting functional capacity is able to recover somewhat. These results add to previous research that suggests both endo‐ and epimicrobiome community structures are in fact influenced by environmental changes (Hollants, Leliaert, Verbruggen, Willems, et al., 2013; Marzinelli et al., 2018), although some research suggests that the environmental impact is only a secondary mechanism and that bacterial associations are strongly determined by host condition and growth stage (Aires et al., 2016; Mancuso et al., 2016; Marzinelli et al., 2015).

The rhizomicrobiome showed the lowest taxonomic and functional resistance to all treatments. However, low resistance to change does not reflect microbial resilience (Shade et al., 2011), and in contrast, the taxonomic and functional resilience of bacterial communities for the individual temperature and nutrient stress treatments indicates a potential recovery of the community structure. The taxonomic and functional resilience of rhizobacterial communities under the combination treatment was near zero, showing no change of the community after recovery period when compared to the community composition poststress. The taxonomic and functional composition was also significantly different from the control. Combination effects of multiple stressors have been shown in some instances to have additive effects (Gouvêa et al., 2017; Strain et al., 2014). Our results suggest that the combination of temperature and nutrient stress may indeed have compounding effects on the rhizomicrobial communities.

Microbial community stability has been defined as either the resistance or resilience to environmental perturbations, or the consistency of the community structure and/or ecological attributes (Pimm, 1984; Worm & Duffy, 2003). However, changes within bacterial community composition do not always result in community instability and could rather indicate a shift to a new stable state (Shade et al., 2012). Holling (1996) summarized two forms of resilience: engineering resilience, which is defined as the ability of an ecological community to recover to the prestress stable state; and ecological resilience, which takes into consideration the community shift from one stable state into a new regime in which the ecosystem is still in equilibrium but different from the initial state. Both types of resilience ensure microbiome stability and aid in maintaining the health of the host organism. Quantitative measures of resistance and resilience can be measured either as compositional stability or functional stability, and research suggests that microbial composition in general does not show high resistance to environmental perturbations (Allison & Martiny, 2008). Our results agree with this notion showing that none of the samples were completely resistant to change. Due to bacterial plasticity and functional redundancy, microbiome stability can be further reinforced, through improved resilience and adaptation (Bashan et al., 2016; Burke et al., 2011; Mandakovic et al., 2018; Shade et al., 2012). Therefore, a community can appear to be compositionally sensitive, but functionally resistant to environmental stress as is seen in this study where the predicted functional capacities proved to be less significantly different from the control when compared to the taxonomic composition. The epi‐ and rhizomicrobiome also demonstrate a higher functional resilience than taxonomic resilience, and their proximity to the external environment may facilitate faster recruitment of functionally equivalent species (Burke et al., 2011). Determining taxa‐function robustness would provide further insight into the ecological resilience of the bacterial community (Eng & Borenstein, 2018). However, functional redundancy is not uniform across different bacterial groups (Griffiths & Philippot, 2013) and the level of functional redundancy within environmental systems remains unclear as the functional capacities of many bacterial groups are yet to be described, or even incorrectly annotated (Allison & Martiny, 2008; Bagheri et al., 2020). Moreover, describing bacterial functional capacities does not elucidate functional expression and individual microbes may modulate their metabolic performance in response to environmental stress (Eng & Borenstein, 2018). Resilience may also be observed for taxa–taxa interactions in which a core set of OTUs maintain crucial functions for bacterial community survival (Mandakovic et al., 2018). Therefore, changes in community composition may be more informative at this stage and may indicate future functional changes (Liu et al., 2018).

It has been suggested that bacteria are able to modulate the responses of the host to environmental changes and can increase host adaptation (Dittami et al., 2016). Bacterial community shifts may facilitate functional changes in order to alleviate environmental stress; therefore, lower taxa‐function robustness may be a mode of adaption utilized for maintaining ecosystem equilibrium (Eng & Borenstein, 2018). Our results show that predicted functions relating to succinoglycan biosynthesis, glucuronokinase, glycogen synthase, maleylacetate reductase, propionate kinase, and DNA‐directed RNA polymerase are found as indicators in response to nutrient stress. Succinoglycan is an exopolysaccharide, which is a key component of algal surface biofilms, which also contains glucuronic acid, glucose, and other dissolved organic carbon (DOC) sources (Decho & Gutierrez, 2017; Reinhold et al., 1994; Sutherland, 2001). Biofilms have multiple functional roles, which including acting as a reservoir of carbon storage, surface adhesion, mediating microbial surface settlement, and chemical defenses (Wahl et al., 2012). Indeed, the significant functional changes observed for the epimicrobiome as a response to an increased nutrient load may be indicative of bacterial mitigation of stress; however, this cannot be confirmed without more detailed analysis of the effects on algal health and the algal bacterial metabolome. Ecological stability is dependent on bacterial competition, and increased diversity introduces metabolic plasticity improving chances for adaptation of species to a particular environmental condition (Coyte et al., 2015). Additionally, the intermediate disturbance hypothesis (IDH) suggests that there is a trade‐off between bacterial competition and community resilience, which supports a higher diversity of bacterial life strategies essential for system stability (Griffiths & Philippot, 2013). Changes to microbial composition introduce new genetic information through horizontal gene flow and by shuffling symbionts in response to environmental changes, and may increase the adaptive capacity of the holobiont (Webster & Reusch, 2017; Ziegler et al., 2017).

Environmental perturbations result in a wide range of downstream effects on microbial communities, and these communities depending on their innate species composition and associations with the host respond in different ways (Shade et al., 2011). Unfavorable changes to bacterial community composition can destabilize community dynamics and have detrimental effects on algal health, either through loss of functional capabilities or chemical defenses (Egan et al., 2013, 2014; Marzinelli et al., 2015; Wahl et al., 2012), which can potentially lead to the introduction of opportunistic pathogens (Campbell et al., 2011; Case et al., 2011). We identified several indicator species associated with the temperature and combination stress that belonged to the Rhodobacteraceae family, which are known to contain notable macroalgal pathogens. This group of bacteria has been shown to contribute to the difference between healthy and diseased red algal tissue (Zozaya‐Valdes et al., 2015), and the abundances on kelp surfaces have been linked to temperature changes (Minich et al., 2018). Pathogenic bacterial species may be naturally present in healthy microbial communities, but only in the decline of algal health and a decrease in algal defenses be allowed to proliferate to detrimental levels. Additionally, pathogenic colonization may occur in stages, and only after a number of stress events does the system shift into a compromised state (Zozaya‐Valdes et al., 2015).

In conclusion, bacterial stability is dependent on complex interactions from various drivers (Orwin & Wardle, 2004). Our results demonstrate that bacterial communities associated with individual morphological niches have distinct responses to different perturbation types and have varying levels of resilience. While we looked exclusively at community responses to environmental change based on compositional changes to microbial structure, changes to functional capacities would provide added value in assessing both functional resilience and taxa‐function robustness (Eng & Borenstein, 2018). Additionally, functional analyses would highlight key metabolic processes associated with the respective abiotic factors (Aires et al., 2016; Burke et al., 2011). This study definitively shows that environmental factors play a role in bacterial community composition. However, it is important to note that this study only assesses the effects of pulse disturbances in the environment and does not address the recovery rates of individual bacterial groups which may be taxa‐specific, which should be investigated further. Additionally, it is still unclear whether these changes observed are a direct response of the bacteria to the abiotic stress, an indirect influence of changes in host condition on the bacterial community, or a complex combination of bacteria–bacteria interactions. Furthermore, the question remains to what extent these changes are necessary for enhancing the host's ability to adapt to environmental change, and if they are not, could this be an indication of host demise?

## CONFLICT OF INTEREST

The authors declare no conflict of interests.

## AUTHOR CONTRIBUTIONS

**Kathryn Lee Morrissey:** Conceptualization (equal); Data curation (lead); Formal analysis (lead); Investigation (lead); Methodology (equal); Project administration (equal); Visualization (lead); Writing‐original draft (lead); Writing‐review & editing (lead). **Ljiljana Iveša:** Data curation (supporting); Investigation (supporting); Project administration (supporting); Resources (supporting); Writing‐review & editing (supporting). **Soria Delva:** Investigation (supporting); Writing‐review & editing (supporting). **Sofie D'Hondt:** Formal analysis (supporting); Investigation (supporting); Methodology (equal). **Anne Willems:** Conceptualization (supporting); Funding acquisition (supporting); Project administration (supporting); Resources (equal); Supervision (supporting); Writing‐review & editing (supporting). **Olivier De Clerck:** Conceptualization (equal); Data curation (supporting); Formal analysis (supporting); Funding acquisition (lead); Investigation (equal); Methodology (supporting); Project administration (equal); Resources (lead); Software (lead); Supervision (lead); Writing‐review & editing (equal).

### OPEN RESEARCH BADGES

This article has earned an Open Data Badge for making publicly available the digitally‐shareable data necessary to reproduce the reported results. The data is available at https://doi.org/10.5061/dryad.sf7m0cg6k.

## Supporting information

Fig S1

Fig S2

Fig S3

Fig S4

Table S1–S8

## Data Availability

DNA sequences: Raw data and necessary inputs are deposited at Dryad (https://doi.org/10.5061/dryad.sf7m0cg6k) and uploaded as a BioProject on NCBI: (Submission ID—SUB7543796; BioProject ID—PRJNA636971).
