# Supplementary material for: Impacts of environmental stress on resistance and resilience of algal‐associated bacterial communities
Source: Ecol Evol. 2021 Oct 6;11(21):15004–19. doi: 10.1002/ece3.8184 (PMC8571626; doi:10.1002/ece3.8184)
Supplement: Supplementary file 5 — Table S1–S8 [file ECE3-11-15004-s001.docx]

| **Table S1. Recorded daily SST for *October 2016.*** | |
| --- | --- |
| **Day** | **Seasurface temperature (°C)** |
| 1 | 21,8 |
| 2 | 21,6 |
| 3 | 21,4 |
| 4 | 21,4 |
| 5 | 21,3 |
| 6 | 21,3 |
| 7 | 21,2 |
| 8 | 21,1 |
| 9 | 20,8 |
| 10 | 20,2 |
| 11 | 20,2 |
| 12 | 20,1 |
| 13 | 19,8 |
| 14 | 19,5 |
| 15 | 19,5 |
| 16 | 19,4 |
| 17 | 19,2 |
| 18 | 19,2 |
| 19 | 19,0 |
| 20 | 19,0 |
| 21 | 19,1 |
| 22 | 18,9 |
| 23 | 18,9 |
| 24 | 18,9 |
| 25 | 18,8 |
| 26 | 18,8 |
| 27 | 18,7 |
| 28 | 18,6 |
| 29 | 18,2 |
| 30 | 18,1 |
| 31 | 17,9 |

| ***Table S2. Summary of environmental conditions.*** | | | | | | |
| --- | --- | --- | --- | --- | --- | --- |
| Environmental parameter | S₀ (pre-stress) | S₁ directly after stress | | | | S₂ (recovery) |
|  | Ambient conditions | Combination stress | Temp Stress | Nutrient stress | Control | Ambient conditions |
| T_max_ (°C) | 18.10 ±1.1 | 25.47 ±0.7 | 23.64 ±0.5 | 17.60 ±0.9 | 17.40 ±0.4 | 16.80 ±0.8 |
| NO_2-_ [µg/l] | 6.71 ±1.2 | 1.03 ±1.1 | 6.72 ±2.0 | 2.58 ±1.1 | 4.21 ±1.9 | 7.22 ±2.6 |
| NO_2-_ : NO_3_ [µg/l] | 32.15 ±11.4 | 6.65 ±4.4 | 10.67 ±4.5 | 14.75 ±1.5 | 12.42 ±4.6 | 28.03 ±16.2 |
| NH_3_ [µg/l] | 129.41 ±8.5 | 14288.85 ±651.4 | 360.44 ±22.8 | 9419.46 ±421.1 | 187.52 ±66.8 | 137.66 ±1.3 |
| PO_4_^3-^ [µg/l] | <1.00 | 3160.66 ±454.8 | 68.40 ±5.0 | 2069.17 ±1650.6 | <1.00 | <1.00 |
| NO_3-_ [µg/l] (calculated) | 26.02 ±13.8 | 6.40 ±4.2 | 4.37 ±2.8 | 5.42 ±0.6 | 8.88 ±4.5 | 21.08 ±10.3 |

| ***Table S3. Permanova analysis results showing the effect of each parameter on the on the Bray-Curtis dissimilarities of OTU numbers for each sample type.*** | | | | | |
| --- | --- | --- | --- | --- | --- |
| Test parameter | Df | Sum Sq | Mean Sq | F value | Pr(>F) |
| Morphological niche | 2 | 1294157 | 647078 | 49.708 | **< 0.001** |
| Timepoint | 1 | 7931 | 7931 | 0.609 | 0.437 |
| Treatment | 3 | 49410 | 16470 | 1.265 | 0.29 |
| Morphological niche:Timepoint | 2 | 11473 | 5737 | 0.441 | 0.645 |
| Morphological niche:Treatment | 6 | 124774 | 20796 | 1.597 | 0.155 |
| Timepoint:Treatment | 3 | 36992 | 12331 | 0.947 | 0.421 |
| Morphological niche:Timepoint:Treatment | 6 | 119404 | 19901 | 1.529 | 0.176 |
| Residuals | 108 | 1405912 | 13018 |  |  |

| ***Table S4. Permanova analysis results showing the effect of each parameter on the Bray-Curtis dissimilarities of the microbial communities.*** | | | | | |
| --- | --- | --- | --- | --- | --- |
| Test parameter | Df | SumOfSqs | R2 | F | Pr(>F) |
| Morphological niche | 4 | 19.752 | 0.30473 | 20.0666 | **0.0001** |
| Experiment number | 1 | 0.277 | 0.00428 | 1.1262 | 0.2692 |
| Timepoint | 1 | 0.466 | 0.00719 | 1.8943 | **0.0433** |
| Treatment | 4 | 1.661 | 0.02563 | 1.6877 | **0.0078** |
| Sequencing run | 1 | 0.317 | 0.00489 | 1.2892 | 0.1844 |
| Morphological niche : Replicate | 4 | 1.788 | 0.02758 | 1.8161 | **0.0039** |
| Morphological niche : Timepoint | 4 | 1.414 | 0.02181 | 1.4365 | **0.0356** |
| Replicate : Timepoint | 1 | 0.301 | 0.00464 | 1.2235 | 0.2082 |
| Morphological niche : Treatment | 12 | 3.104 | 0.04789 | 1.0511 | 0.3183 |
| Replicate : Treatment | 4 | 0.947 | 0.01461 | 0.9619 | 0.5238 |
| Timepoint : Treatment | 3 | 0.976 | 0.01505 | 1.3218 | 0.0967 |
| Replicate : Sequencing run | 1 | 0.365 | 0.00563 | 1.4838 | 0.0882 |
| Treatment : Sequencing run | 2 | 0.621 | 0.00957 | 1.2609 | 0.1003 |
| Morphological niche : Replicate : Timepoint | 4 | 1.131 | 0.01745 | 1.1488 | 0.2145 |
| Morphological niche : Replicate : Treatment | 12 | 2.586 | 0.0399 | 0.8758 | 0.8552 |
| Morphological niche : Timepoint : Treatment | 9 | 2.407 | 0.03714 | 1.087 | 0.2531 |
| Replicate : Timepoint : Treatment | 3 | 0.599 | 0.00925 | 0.8119 | 0.7832 |
| Morphological niche : Replicate : Timepoint : Treatment | 9 | 1.989 | 0.03068 | 0.898 | 0.7695 |
| Residual | 98 | 24.116 | 0.37206 |  |  |
| Total | 177 | 64.817 | 1 |  |  |

| ***Table S5. Comparisons of bacterial taxonomic composition under nutrient, temperature and the combination stress treatments in response to the control, using General Linear Hypotheses of Bray-Curtis dissimilarity metrics corrected by the single step method*** | | | | | | | | | | | | | | | | |  |
| --- | --- | --- | --- | --- | --- | --- | --- | --- | --- | --- | --- | --- | --- | --- | --- | --- | --- |
| Day 0 – before stress | | | | | | | | | | | | | | | | |  |
|  | Endomicrobiome | | | |  | Epimicrobiome | | | |  | Rhizomicrobiome | | | | | | |
| Treatment | Estimate | Std. Error | t value | Pr(>\|t\|) |  | Estimate | Std. Error | t value | Pr(>\|t\|) |  | Estimate | Std. Error | t value | Pr(>\|t\|) | |  |  |
| Nutrients | 0.06360 | 0.10269 | 0.619 | 0.9978 |  | 0.24362 | 0.10052 | 2.424 | 0.16499 |  | -0.04215 | 0.04881 | -0.864 | 0.98061 | |  |  |
| Temperature | 0.12074 | 0.10269 | 1.176 | 0.8770 |  | 0.13064 | 0.11239 | 1.162 | 0.89243 |  | -0.11294 | 0.04881 | -2.314 | 0.21420 | |  |  |
| Temperature + Nutrients | 0.03266 | 0.10269 | 0.318 | 1.0000 |  | 0.15005 | 0.10052 | 1.493 | 0.69904 |  | -0.08934 | 0.04881 | -1.830 | 0.47363 | |  |  |
| Day 3 – directly after stress | | | | | | | | | | | | | | |  | |  |
|  | Endomicrobiome | | | |  | Epimicrobiome | | | |  | Rhizomicrobiome | | | | |  | |
| Treatment | Estimate | Std. Error | t value | Pr(>\|t\|) |  | Estimate | Std. Error | t value | Pr(>\|t\|) |  | Estimate | Std. Error | t value | Pr(>\|t\|) | |  |  |
| Nutrients | 0.04787 | 0.06258 | 0.765 | 0.9504 |  | 0.32678 | 0.07455 | 4.383 | **0.00114** |  | 0.19989 | 0.04881 | 4.095 | **0.00332** | |  |  |
| Temperature | 0.13618 | 0.06495 | 2.097 | 0.1996 |  | 0.16852 | 0.07455 | 2.261 | 0.22688 |  | 0.24463 | 0.04566 | 5.358 | **< 0.001** | |  |  |
| Temperature + Nutrients | 0.12702 | 0.06495 | 1.956 | 0.2594 |  | 0.14169 | 0.07786 | 1.820 | 0.47170 |  | 0.19447 | 0.04881 | 3.984 | **0.00467** | |  |  |
| Day 12 – after the recovery period | | | | | | | | | | | | | | | | |  |
|  | Endomicrobiome | | | |  | Epimicrobiome | | | |  | Rhizomicrobiome | | | | | | |
| Treatment | Estimate | Std. Error | t value | Pr(>\|t\|) |  | Estimate | Std. Error | t value | Pr(>\|t\|) |  | Estimate | Std. Error | t value | Pr(>\|t\|) | |  |  |
| Nutrients | 0.023150 | 0.09185 | 2.520 | 0.0823 |  | 0.30087 | 0.10052 | 2.993 | **0.04652** |  | 0.10847 | 0.04366 | 2.485 | 0.15402 | |  |  |
| Temperature | 0.26698 | 0.09185 | 2.907 | **0.0331** |  | 0.37085 | 0.10052 | 3.689 | **0.00776** |  | 0.13620 | 0.04566 | 3.120 | **0.03856** | |  |  |
| Temperature + Nutrients | 0.19671 | 0.09185 | 2.142 | 0.1829 |  | 0.20855 | 0.10052 | 2.075 | 0.31666 |  | 0.20680 | 0.04366 | 4.737 | **< 0.001** | |  |  |

| ***Table S6. Summary of the indicator species analysis identifying significant OTUs (species) associated to the differences observed for each morphological niche between treatments at day 3 (directly after stress) and day 12 (after the recovery period).*** | | | | | | | | | | | |
| --- | --- | --- | --- | --- | --- | --- | --- | --- | --- | --- | --- |
|  |  |  |  |  |  |  |  | Treatment association | | | |
| OTU | phylum | class | order | family | genus | Indicator statistic | p.value | Control | Nutrients | Temperature | Temperature + Nutrients |
| **Endomicrobiome** | | | | | | | | | | | |
| **Day 3 - directly after stress** | | | | | | | | | | | |
| Otu240 | Proteobacteria | Gammaproteobacteria | Vibrionales | Vibrionaceae | unclassified | 0.762 | 0.0368 |  |  | * | * |
| Otu880 | Proteobacteria | Gammaproteobacteria | Enterobacteriales | Enterobacteriaceae | unclassified | 0.723 | 0.0319 |  | * |  |  |
| Otu1278 | Proteobacteria | Gammaproteobacteria | Xanthomonadales | Xanthomonadaceae | unclassified | 0.655 | 0.0407 |  | * |  |  |
| Otu1589 | Proteobacteria | Alphaproteobacteria | Rhodobacterales | Rhodobacteraceae | unclassified | 0.717 | 0.0479 |  |  | * |  |
| Otu1591 | Proteobacteria | Alphaproteobacteria | Rhodobacterales | Rhodobacteraceae | unclassified | 0.714 | 0.0206 |  |  | * |  |
| Otu4180 | Proteobacteria | Alphaproteobacteria | Rhodobacterales | Rhodobacteraceae | unclassified | 0.707 | 0.0268 |  |  | * |  |
| Otu4699 | Proteobacteria | Alphaproteobacteria | Rhodobacterales | Rhodobacteraceae | unclassified | 0.707 | 0.0286 |  |  | * |  |
| Otu2038 | Proteobacteria | Gammaproteobacteria | Oceanospirillales | unclassified | unclassified | 0.707 | 0.0253 |  |  | * |  |
| **Day 12 - after the recovery period** | | | | | | | | | | | |
| Otu144 | Proteobacteria | Gammaproteobacteria | unclassified | unclassified | unclassified | 0.972 | 0.038 | * |  |  |  |
| **Epimicrobiome** | | | | | | | | | | | |
| **Day 3 - directly after stress** | | | | | | | | | | | |
| Otu5829 | Bacteroidetes | unclassified | unclassified | unclassified | unclassified | 0.651 | 0.0449 |  |  |  | * |
| Otu82 | Firmicutes | unclassified | unclassified | unclassified | unclassified | 0.753 | 0.0291 |  |  |  | * |
| Otu6022 | Proteobacteria | Alphaproteobacteria | Rhodobacterales | Rhodobacteraceae | unclassified | 0.724 | 0.023 |  |  |  | * |
| Otu232 | Proteobacteria | Gammaproteobacteria | unclassified | unclassified | unclassified | 0.74 | 0.0304 |  |  |  | * |
| Otu608 | Proteobacteria | Gammaproteobacteria | unclassified | unclassified | unclassified | 0.701 | 0.0243 |  |  |  | * |
| Otu6691 | unclassified | unclassified | unclassified | unclassified | unclassified | 0.732 | 0.0302 | * |  |  | * |
| Otu7113 | Proteobacteria | Gammaproteobacteria | Enterobacteriales | Enterobacteriaceae | unclassified | 0.765 | 0.023 |  | * |  |  |
| Otu363 | Bacteroidetes | Flavobacteriia | Flavobacteriales | Flavobacteriaceae | unclassified | 0.734 | 0.0342 | * |  |  |  |
| Otu48 | Firmicutes | Bacilli | Bacillales | unclassified | unclassified | 0.707 | 0.0396 |  |  |  |  |
| Otu407 | Firmicutes | Clostridia | Clostridiales | unclassified | unclassified | 0.697 | 0.0495 |  |  | * |  |
| Otu528 | Firmicutes | Clostridia | Clostridiales | unclassified | unclassified | 0.775 | 0.0122 | * |  |  |  |
| Otu312 | Proteobacteria | Alphaproteobacteria | Rhizobiales | Cohaesibacteraceae | Cohaesibacter | 0.753 | 0.025 | * |  |  |  |
| Otu466 | Proteobacteria | Alphaproteobacteria | Rhodobacterales | Rhodobacteraceae | Celeribacter | 0.739 | 0.0449 | * |  | * |  |
| Otu4188 | Proteobacteria | Alphaproteobacteria | Rhodobacterales | Rhodobacteraceae | unclassified | 0.705 | 0.0315 | * |  |  |  |
| Otu238 | Proteobacteria | Alphaproteobacteria | unclassified | unclassified | unclassified | 0.708 | 0.0345 | * |  |  |  |
| Otu3011 | Proteobacteria | Deltaproteobacteria | Desulfobacterales | Desulfobulbaceae | unclassified | 0.759 | 0.0249 | * |  |  |  |
| **Day 12 - after the recovery period** | | |  |  |  |  |  |  |  |  |  |
| Otu101 | Proteobacteria | Alphaproteobacteria | Rhodobacterales | Rhodobacteraceae | unclassified | 0.985 | 0.0131 |  |  | * | * |
| Otu2969 | Proteobacteria | Alphaproteobacteria | Rhodobacterales | Rhodobacteraceae | unclassified | 0.913 | 0.046 |  |  | * | * |
| Otu401 | Proteobacteria | Alphaproteobacteria | Rhodobacterales | Rhodobacteraceae | unclassified | 0.988 | 0.0125 |  |  | * | * |
| Otu64 | Proteobacteria | Alphaproteobacteria | Rhodobacterales | Rhodobacteraceae | Jannaschia | 0.909 | 0.039 |  |  | * | * |
| Otu71 | Proteobacteria | Alphaproteobacteria | Rhodobacterales | Rhodobacteraceae | unclassified | 0.913 | 0.045 |  |  | * | * |
| Otu2736 | Proteobacteria | Alphaproteobacteria | Rhodobacterales | Rhodobacteraceae | unclassified | 1 | 0.017 |  |  |  | * |
| Otu180 | Proteobacteria | Deltaproteobacteria | Desulfobacterales | Desulfobulbaceae | unclassified | 0.957 | 0.0396 |  |  | * | * |
| Otu587 | Proteobacteria | Deltaproteobacteria | Desulfobacterales | Desulfobulbaceae | unclassified | 0.904 | 0.0474 |  |  | * | * |
| Otu4355 | Proteobacteria | Deltaproteobacteria | Desulfobacterales | Desulfobulbaceae | unclassified | 1 | 0.017 |  |  |  | * |
| Otu63 | Proteobacteria | Epsilonproteobacteria | Campylobacterales | Helicobacteraceae | Sulfurovum | 0.893 | 0.046 |  |  | * | * |
| Otu5627 | Proteobacteria | unclassified | unclassified | unclassified | unclassified | 0.913 | 0.045 |  |  | * | * |
| Otu1178 | Proteobacteria | Alphaproteobacteria | Rhodobacterales | Rhodobacteraceae | Litoreibacter | 1 | 0.0067 |  |  | ** | ** |
| Otu189 | Proteobacteria | Alphaproteobacteria | Rhodobacterales | Rhodobacteraceae | unclassified | 0.966 | 0.0075 |  |  | ** | ** |
| Otu2546 | Proteobacteria | Alphaproteobacteria | unclassified | unclassified | unclassified | 1 | 0.0067 |  |  | ** | ** |
| Otu20 | Proteobacteria | Deltaproteobacteria | Desulfobacterales | Desulfobulbaceae | unclassified | 1 | 0.0067 |  |  | ** | ** |
| Otu214 | Proteobacteria | unclassified | unclassified | unclassified | unclassified | 1 | 0.0067 |  |  | ** | ** |
| Otu40 | Proteobacteria | Gammaproteobacteria | Enterobacteriales | Enterobacteriaceae | unclassified | 0.972 | 0.0127 | * | * |  |  |
| Otu209 | Proteobacteria | Gammaproteobacteria | Pseudomonadales | Moraxellaceae | Acinetobacter | 0.913 | 0.0487 | * | * |  |  |
| Otu439 | Actinobacteria | Actinobacteria | Acidimicrobiales | Acidimicrobiaceae | Ilumatobacter | 0.897 | 0.0457 | * |  | * |  |
| Otu58 | Bacteroidetes | Cytophagia | Cytophagales | unclassified | unclassified | 0.916 | 0.0368 |  |  | * |  |
| Otu68 | Firmicutes | Clostridia | Clostridiales | unclassified | unclassified | 0.96 | 0.0195 |  |  | * |  |
| Otu102 | Proteobacteria | Alphaproteobacteria | Rhodobacterales | Rhodobacteraceae | Donghicola | 0.949 | 0.0363 |  |  | * |  |
| Otu44 | Proteobacteria | Alphaproteobacteria | Sphingomonadales | Sphingomonadaceae | Sphingorhabdus | 1 | 0.0195 |  |  | * |  |
| Otu451 | Proteobacteria | Deltaproteobacteria | Desulfobacterales | Desulfobulbaceae | unclassified | 1 | 0.0195 |  |  | * |  |
| Otu54 | Proteobacteria | Gammaproteobacteria | Chromatiales | unclassified | unclassified | 0.875 | 0.0396 |  |  | * |  |
| Otu258 | Proteobacteria | Gammaproteobacteria | incertae_sedis | unclassified | unclassified | 0.925 | 0.0254 |  |  | * |  |
| Otu1143 | Proteobacteria | Gammaproteobacteria | unclassified | unclassified | unclassified | 1 | 0.0195 |  |  | * |  |
| Otu347 | Proteobacteria | Gammaproteobacteria | unclassified | unclassified | unclassified | 0.961 | 0.0368 |  |  | * |  |
| Otu7657 | Proteobacteria | Gammaproteobacteria | unclassified | unclassified | unclassified | 1 | 0.0195 |  |  | * |  |
| Otu4189 | Proteobacteria | unclassified | unclassified | unclassified | unclassified | 0.933 | 0.0363 |  |  | * |  |
| **Rhizomicrobiome** | | | | | | | | | | | |
| **Day 3 - directly after stress** | | | | | | | | | | | |
| Otu1598 | Bacteroidetes | Flavobacteriia | Flavobacteriales | Flavobacteriaceae | unclassified | 0.943 | 0.0217 | * | * |  | * |
| Otu149 | Proteobacteria | Gammaproteobacteria | unclassified | unclassified | unclassified | 0.937 | 0.0367 |  | * | * | * |
| Otu371 | Firmicutes | Clostridia | Clostridiales | unclassified | unclassified | 0.913 | 0.0133 | * |  |  | * |
| Otu284 | Proteobacteria | Alphaproteobacteria | Rhodobacterales | Rhodobacteraceae | unclassified | 0.875 | 0.0395 | * |  |  | * |
| Otu1537 | Proteobacteria | Alphaproteobacteria | Rhodobacterales | Rhodobacteraceae | Marivita | 0.913 | 0.0179 | * | * |  |  |
| Otu44 | Proteobacteria | Alphaproteobacteria | Sphingomonadales | Sphingomonadaceae | Sphingorhabdus | 0.886 | 0.0444 |  | * |  |  |
| Otu2588 | Proteobacteria | Deltaproteobacteria | Desulfobacterales | Desulfobulbaceae | unclassified | 0.904 | 0.0452 |  | * | * |  |
| Otu53 | Actinobacteria | Actinobacteria | Acidimicrobiales | Iamiaceae | Aquihabitans | 0.959 | 0.0074 |  | ** |  |  |
| Otu1330 | Bacteroidetes | unclassified | unclassified | unclassified | unclassified | 0.866 | 0.0249 |  |  | * |  |
| Otu473 | Bacteroidetes | unclassified | unclassified | unclassified | unclassified | 0.822 | 0.046 |  |  | * |  |
| Otu60 | Proteobacteria | Deltaproteobacteria | Desulfobacterales | Desulfobacteraceae | unclassified | 0.898 | 0.0348 | * |  | * |  |
| Otu922 | Proteobacteria | Deltaproteobacteria | Desulfobacterales | Desulfobacteraceae | unclassified | 0.89 | 0.0387 | * |  | * |  |
| Otu357 | Proteobacteria | Deltaproteobacteria | Desulfobacterales | Desulfobacteraceae | unclassified | 0.866 | 0.0226 |  |  | * |  |
| Otu888 | Proteobacteria | Deltaproteobacteria | Desulfobacterales | Desulfobacteraceae | unclassified | 0.866 | 0.0249 |  |  | * |  |
| Otu5657 | Proteobacteria | Deltaproteobacteria | Desulfobacterales | Desulfobulbaceae | unclassified | 0.866 | 0.0243 |  |  | * |  |
| Otu308 | Proteobacteria | Deltaproteobacteria | Desulfobacterales | unclassified | unclassified | 0.95 | 0.0253 | * |  | * |  |
| Otu4851 | unclassified | unclassified | unclassified | unclassified | unclassified | 0.866 | 0.0243 |  |  | * |  |
| **Day 12 - after the recovery period** | | | | | | | | | | | |
| Otu804 | Proteobacteria | Deltaproteobacteria | Desulfobacterales | Desulfobulbaceae | unclassified | 0.988 | 0.024 |  | * | * | * |
| Otu451 | Proteobacteria | Deltaproteobacteria | Desulfobacterales | Desulfobulbaceae | unclassified | 0.99 | 0.0334 | * | * |  | * |
| Otu813 | Proteobacteria | Deltaproteobacteria | Desulfobacterales | Desulfobulbaceae | Desulfobulbus | 0.816 | 0.0315 |  |  | * | * |
| Otu819 | Proteobacteria | Gammaproteobacteria | unclassified | unclassified | unclassified | 0.949 | 0.031 | * |  | * | * |
| Otu318 | unclassified | unclassified | unclassified | unclassified | unclassified | 0.901 | 0.0145 |  |  | * | * |
| Otu2598 | Proteobacteria | Deltaproteobacteria | Desulfobacterales | Desulfobulbaceae | unclassified | 0.993 | 0.0096 |  | ** | ** | ** |
| Otu5736 | Proteobacteria | Deltaproteobacteria | Desulfobacterales | Desulfobulbaceae | unclassified | 0.913 | 0.0067 |  |  | ** | ** |
| Otu1296 | Proteobacteria | unclassified | unclassified | unclassified | unclassified | 0.901 | 0.0063 |  |  |  | ** |
| Otu328 | Bacteroidetes | Flavobacteriia | Flavobacteriales | Flavobacteriaceae | Lutibacter | 0.895 | 0.0467 |  | * | * |  |
| Otu637 | Proteobacteria | Alphaproteobacteria | unclassified | unclassified | unclassified | 0.953 | 0.0225 | * | * | * |  |
| Otu617 | Proteobacteria | Deltaproteobacteria | Desulfobacterales | Desulfobulbaceae | unclassified | 0.896 | 0.0365 | * | * |  |  |
| Otu6320 | Proteobacteria | Deltaproteobacteria | Desulfobacterales | Desulfobulbaceae | unclassified | 0.866 | 0.0291 |  | * |  |  |
| Otu8057 | Proteobacteria | Deltaproteobacteria | Desulfobacterales | Desulfobulbaceae | unclassified | 0.818 | 0.0382 |  | * |  |  |
| Otu2113 | Proteobacteria | unclassified | unclassified | unclassified | unclassified | 0.925 | 0.0156 |  | * | * |  |
| Otu5637 | Proteobacteria | unclassified | unclassified | unclassified | unclassified | 0.866 | 0.029 |  | * |  |  |
| Otu8521 | Proteobacteria | unclassified | unclassified | unclassified | unclassified | 0.866 | 0.0291 |  | * |  |  |
| Otu699 | unclassified | unclassified | unclassified | unclassified | unclassified | 0.839 | 0.0357 |  | * |  |  |
| Otu214 | Proteobacteria | unclassified | unclassified | unclassified | unclassified | 0.961 | 0.0019 |  | ** |  |  |
| Otu2406 | unclassified | unclassified | unclassified | unclassified | unclassified | 0.926 | 0.0096 |  | ** | ** |  |
| Otu689 | Bacteroidetes | Bacteroidia | Bacteroidales | Prolixibacteraceae | unclassified | 0.835 | 0.029 | * |  |  |  |
| Otu3299 | Bacteroidetes | Flavobacteriia | Flavobacteriales | Flavobacteriaceae | Winogradskyella | 0.832 | 0.0382 | * |  |  |  |
| Otu297 | Bacteroidetes | Sphingobacteriia | Sphingobacteriales | Saprospiraceae | unclassified | 0.938 | 0.0111 | * |  |  |  |
| Otu473 | Bacteroidetes | unclassified | unclassified | unclassified | unclassified | 0.943 | 0.0086 |  |  | ** |  |
| Otu1012 | Bacteroidetes | unclassified | unclassified | unclassified | unclassified | 0.866 | 0.0295 | * |  |  |  |
| Otu1161 | Firmicutes | unclassified | unclassified | unclassified | unclassified | 0.866 | 0.0296 | * |  |  |  |
| Otu296 | Proteobacteria | Alphaproteobacteria | Sphingomonadales | Erythrobacteraceae | Erythrobacter | 0.909 | 0.0176 | * |  | * |  |
| Otu600 | Proteobacteria | Deltaproteobacteria | Bdellovibrionales | Bacteriovoracaceae | unclassified | 0.866 | 0.0295 | * |  |  |  |
| Otu5612 | Proteobacteria | Deltaproteobacteria | Desulfobacterales | Desulfobacteraceae | unclassified | 0.866 | 0.0295 | * |  |  |  |
| Otu3324 | Proteobacteria | Deltaproteobacteria | Desulfobacterales | Desulfobulbaceae | Desulfobulbus | 0.866 | 0.0229 |  |  | * |  |
| Otu168 | Proteobacteria | Gammaproteobacteria | Alteromonadales | Pseudoalteromonadaceae | Pseudoalteromonas | 0.813 | 0.0407 | * |  |  |  |
| Otu54 | Proteobacteria | Gammaproteobacteria | Chromatiales | unclassified | unclassified | 1 | 0.0024 | ** |  |  |  |
| Otu14 | Proteobacteria | Gammaproteobacteria | Oceanospirillales | Halomonadaceae | Cobetia | 0.98 | 0.0147 | * |  |  |  |

| ***Table S7. Comparisons of the significant changes in the predicted functional structure under nutrient, temperature and the combination stress treatments in response to the control, using General Linear Hypotheses of Bray-Curtis dissimilarity metrics corrected by the single step method*** | | | | | | | | | | | | | | | | |  |
| --- | --- | --- | --- | --- | --- | --- | --- | --- | --- | --- | --- | --- | --- | --- | --- | --- | --- |
| Day 0 – before stress | | | | | | | | | | | | | | | | |  |
|  | Endomicrobiome | | | |  | Epimicrobiome | | | |  | Rhizomicrobiome | | | | | | |
| Treatment | Estimate | Std. Error | t value | Pr(>\|t\|) |  | Estimate | Std. Error | t value | Pr(>\|t\|) |  | Estimate | Std. Error | t value | Pr(>\|t\|) | |  |  |
| Nutrients | -0.019364 | 0.099421 | -0.195 | 1 |  | 0.08882 | 0.09256 | 0.96 | 0.9616 |  | 0.008655 | 0.031854 | 0.272 | 1 | |  |  |
| Temperature | 0.114622 | 0.099421 | 1.153 | 0.888 |  | 0.01592 | 0.08279 | 0.192 | 1 |  | 0.031536 | 0.031854 | 0.99 | 0.9537 | |  |  |
| Temperature + Nutrients | 0.209727 | 0.099421 | 2.109 | 0.288 |  | -0.05826 | 0.08279 | -0.704 | 0.9952 |  | -0.013611 | 0.031854 | -0.427 | 0.9999 | |  |  |
| Day 3 – directly after stress | | | | | | | | | | | | | | |  | |  |
|  | Endomicrobiome | | | |  | Epimicrobiome | | | |  | Rhizomicrobiome | | | | |  | |
| Treatment | Estimate | Std. Error | t value | Pr(>\|t\|) |  | Estimate | Std. Error | t value | Pr(>\|t\|) |  | Estimate | Std. Error | t value | Pr(>\|t\|) | |  |  |
| Nutrients | 0.055387 | 0.060592 | 0.914 | 0.968 |  | 0.18854 | 0.0614 | 3.071 | **0.0385** |  | 0.012231 | 0.031854 | 0.384 | 1 | |  |  |
| Temperature | 0.03388 | 0.062879 | 0.539 | 0.999 |  | 0.0401 | 0.0614 | 0.653 | 0.9973 |  | 0.056232 | 0.029797 | 1.887 | 0.435 | |  |  |
| Temperature + Nutrients | -0.001646 | 0.062879 | -0.026 | 1 |  | 0.10694 | 0.06413 | 1.668 | 0.5766 |  | 0.025497 | 0.031854 | 0.8 | 0.9877 | |  |  |
| Day 12 – after the recovery period | | | | | | | | | | | | | | | | |  |
|  | Endomicrobiome | | | |  | Epimicrobiome | | | |  | Rhizomicrobiome | | | | | | |
| Treatment | Estimate | Std. Error | t value | Pr(>\|t\|) |  | Estimate | Std. Error | t value | Pr(>\|t\|) |  | Estimate | Std. Error | t value | Pr(>\|t\|) | |  |  |
| Nutrients | 0.183072 | 0.088925 | 2.059 | 0.314 |  | 0.17433 | 0.08279 | 2.106 | 0.3002 |  | 0.01583 | 0.029797 | 0.531 | 0.9995 | |  |  |
| Temperature | 0.211509 | 0.088925 | 2.379 | 0.173 |  | 0.09279 | 0.08279 | 1.121 | 0.9101 |  | 0.030375 | 0.029797 | 1.019 | 0.9455 | |  |  |
| Temperature + Nutrients | 0.175815 | 0.088925 | 1.977 | 0.36 |  | 0.12539 | 0.08279 | 1.515 | 0.6839 |  | 0.092011 | 0.029797 | 3.088 | **0.0422** | |  |  |

| ***Table S8. Summary of the indicator species analysis identifying significant KOs (predicted functions) associated to the differences observed for each morphological niche between treatments at day 3 (directly after stress) and day 12 (after the recovery period).*** | | | | | | | |
| --- | --- | --- | --- | --- | --- | --- | --- |
|  |  |  |  | Treatment association | | | |
| KO | Function | Indicator statistic | p.value | Control | Nutrients | Temperature | Temperature + Nutrients |
| **Endomicrobiome** | | | | | | | |
| **Day 3 - directly after stress** | | | | | | | |
| K14470 | 2-methylfumaryl-CoA isomerase [EC:5.4.1.3] | 0.927 | 0.0337 |  | * | * | * |
| K05797 | 4-cresol dehydrogenase (hydroxylating) flavoprotein subunit [EC:1.17.99.1] | 0.812 | 0.0428 |  | * | * |  |
| K10211 | 4,4'-diaponeurosporenoate glycosyltransferase [EC:2.4.1.-] | 0.902 | 0.0219 |  | * | * | * |
| K12269 | accessory secretory protein Asp2 | 0.758 | 0.0482 |  | * | * |  |
| K12292 | ATP-binding cassette, subfamily C, bacterial, competence factor transporting protein [EC:3.4.22.-] | 0.764 | 0.0239 |  | * | * |  |
| K10910 | autoinducer 2-binding periplasmic protein LuxP | 0.975 | 0.0405 |  | * | * | * |
| K01060 | cephalosporin-C deacetylase [EC:3.1.1.41] | 0.84 | 0.0423 | * | * | * |  |
| K03333 | cholesterol oxidase [EC:1.1.3.6] | 0.958 | 0.0491 | * | * | * |  |
| K19073 | divinyl chlorophyllide a 8-vinyl-reductase [EC:1.3.1.75] | 0.924 | 0.0183 |  | * | * | * |
| K13797 | DNA-directed RNA polymerase subunit beta-beta' [EC:2.7.7.6] | 0.707 | 0.0388 |  | * |  |  |
| K16559 | endo-1,3-1,4-beta-glycanase ExoK [EC:3.2.1.-] | 0.859 | 0.0198 |  | * |  |  |
| K02639 | ferredoxin | 0.906 | 0.0335 |  | * | * | * |
| K00118 | glucose-fructose oxidoreductase [EC:1.1.99.28] | 0.911 | 0.0273 |  | * | * | * |
| K00693 | glycogen synthase [EC:2.4.1.11] | 0.779 | 0.0294 |  | * |  |  |
| K10212 | glycosyl-4,4'-diaponeurosporenoate acyltransferase [EC:2.3.1.-] | 0.895 | 0.0298 |  | * | * | * |
| K07215 | heme oxygenase (biliverdin-IX-beta and delta-forming) [EC:1.14.99.58] | 0.978 | 0.0245 | * | * | * |  |
| K19350 | lincosamide and streptogramin A transport system ATP-binding/permease protein | 0.841 | 0.0155 |  | * | * |  |
| K08692 | malate-CoA ligase subunit alpha [EC:6.2.1.9] | 0.903 | 0.0294 |  | * | * | * |
| K14067 | malate-CoA ligase subunit beta [EC:6.2.1.9] | 0.9 | 0.0362 |  | * | * | * |
| K00217 | maleylacetate reductase [EC:1.3.1.32] | 0.664 | 0.0466 |  | * |  |  |
| K09819 | manganese/iron transport system permease protein | 0.94 | 0.0152 |  | * | * | * |
| K01461 | N-acyl-D-glutamate deacylase [EC:3.5.1.82] | 0.906 | 0.0415 |  | * | * | * |
| K01003 | oxaloacetate decarboxylase [EC:4.1.1.3] | 0.949 | 0.0332 | * | * | * |  |
| K09459 | phosphonopyruvate decarboxylase [EC:4.1.1.82] | 0.754 | 0.0455 |  | * | * |  |
| K18067 | phthalate 4,5-cis-dihydrodiol dehydrogenase [EC:1.3.1.64] | 0.903 | 0.0188 |  | * | * | * |
| K19697 | propionate kinase [EC:2.7.2.15] | 0.707 | 0.0353 |  | * |  |  |
| K04712 | sphingolipid 4-desaturase/C4-monooxygenase [EC:1.14.19.17 1.14.18.5] | 0.799 | 0.0437 |  | * | * |  |
| K06045 | squalene-hopene/tetraprenyl-beta-curcumene cyclase [EC:5.4.99.17 4.2.1.129] | 0.847 | 0.0423 |  | * | * |  |
| K16562 | succinoglycan biosynthesis protein ExoW [EC:2.4.-.-] | 0.785 | 0.0184 |  | * |  |  |
| K00917 | tagatose 6-phosphate kinase [EC:2.7.1.144] | 0.902 | 0.0112 |  | * | * | * |
| K19519 | transforming growth factor-beta-induced protein | 0.764 | 0.0366 |  | * | * |  |
| K19661 | two-component system, NtrC family, sensor histidine kinase HupT/HoxJ [EC:2.7.13.3] | 0.874 | 0.0144 |  | * | * | * |
| K11898 | type VI secretion system protein ImpE | 0.955 | 0.0375 |  | * | * | * |
| K08068 | UDP-N-acetylglucosamine 2-epimerase (hydrolysing) [EC:3.2.1.183] | 0.923 | 0.0297 |  | * | * | * |
| K09155 | uncharacterized protein | 0.938 | 0.0137 |  | * | * |  |
| K09144 | uncharacterized protein | 0.832 | 0.0223 |  | * |  |  |
| K00996 | undecaprenyl-phosphate galactose phosphotransferase [EC:2.7.8.6] | 0.904 | 0.0352 | * | * | * |  |
| K01198 | xylan 1,4-beta-xylosidase [EC:3.2.1.37] | 0.94 | 0.0465 |  | * | * | * |
| K07260 | zinc D-Ala-D-Ala carboxypeptidase [EC:3.4.17.14] | 0.966 | 0.0341 | * | * | * |  |
| K16927 | energy-coupling factor transport system substrate-specific component | 0.957 | 0.0044 |  | ** | ** | ** |
| K16190 | glucuronokinase [EC:2.7.1.43] | 0.816 | 0.0063 |  | ** |  |  |
| K00613 | glycine amidinotransferase [EC:2.1.4.1] | 0.961 | 0.0048 |  | ** | ** | ** |
| K16555 | succinoglycan biosynthesis protein ExoO [EC:2.4.-.-] | 0.93 | 0.0047 |  | ** |  |  |
| K06873 | uncharacterized protein | 0.941 | 0.007 |  | ** | ** |  |
| K14956 | 6 kDa early secretory antigenic target | 0.775 | 0.0137 | * |  |  |  |
| K00842 | aminotransferase [EC:2.6.1.-] | 0.721 | 0.0286 | * |  |  |  |
| K18701 | arsenate-mycothiol transferase [EC:2.8.4.2] | 0.743 | 0.0333 | * |  |  |  |
| K14698 | ATP-binding cassette, subfamily B, bacterial IrtA [EC:3.6.3.-] | 0.741 | 0.0286 | * |  |  |  |
| K14699 | ATP-binding cassette, subfamily B, bacterial IrtB [EC:3.6.3.-] | 0.759 | 0.0141 | * |  |  |  |
| K18895 | ATP-binding cassette, subfamily B, salmochelin/enterobactin exporter | 0.705 | 0.0296 | * |  |  |  |
| K11953 | bicarbonate transport system ATP-binding protein [EC:3.6.3.-] | 0.769 | 0.0305 |  |  |  | * |
| K13381 | bifunctional chitinase/lysozyme [EC:3.2.1.14 3.2.1.17] | 0.929 | 0.0322 | * |  | * | * |
| K07291 | CDP-L-myo-inositol myo-inositolphosphotransferase [EC:2.7.8.34] | 0.789 | 0.0242 |  |  |  |  |
| K18589 | dihydrofolate reductase (trimethoprim resistance protein) [EC:1.5.1.3] | 0.775 | 0.0122 | * |  |  |  |
| K18288 | itaconate CoA-transferase [EC:2.8.3.-] | 0.817 | 0.0183 |  |  | * |  |
| K10215 | monooxygenase [EC:1.14.13.-] | 0.775 | 0.0137 | * |  |  |  |
| K13380 | NADH-quinone oxidoreductase subunit B/C/D [EC:1.6.5.3] | 0.819 | 0.0307 |  |  | * |  |
| K12227 | TraL protein | 0.775 | 0.0122 | * |  |  |  |
| **Day 12 - after the recovery period** | |  |  |  |  |  |  |
| K18887 | ATP-binding cassette, subfamily B, multidrug efflux pump | 0.921 | 0.0208 |  | * |  |  |
| K18888 | ATP-binding cassette, subfamily B, multidrug efflux pump | 0.913 | 0.0467 | * | * |  |  |
| K15780 | bifunctional protein TilS/HprT [EC:6.3.4.19 2.4.2.8] | 0.957 | 0.0408 |  | * |  |  |
| K12070 | conjugal transfer pilus assembly protein TraI | 1 | 0.0208 |  | * |  |  |
| K01884 | cysteinyl-tRNA synthetase, unknown class [EC:6.1.1.16] | 1 | 0.0208 |  | * |  |  |
| K17810 | D-aspartate ligase [EC:6.3.1.12] | 1 | 0.0208 |  | * |  |  |
| K06608 | DeoR family transcriptional regulator, myo-inositol catabolism operon repressor | 1 | 0.0208 |  | * |  |  |
| K19350 | lincosamide and streptogramin A transport system ATP-binding/permease protein | 1 | 0.0208 |  | * |  |  |
| K18094 | membrane fusion protein, multidrug efflux system | 1 | 0.0208 |  | * |  |  |
| K18009 | meso-butanediol dehydrogenase / (S,S)-butanediol dehydrogenase / diacetyl reductase [EC:1.1.1.- 1.1.1.76 1.1.1.304] | 0.998 | 0.0364 | * | * |  | * |
| K12281 | MSHA biogenesis protein MshK | 0.996 | 0.0306 | * | * |  |  |
| K12284 | MSHA biogenesis protein MshN | 1 | 0.0182 | * | * |  | * |
| K00477 | phytanoyl-CoA hydroxylase [EC:1.14.11.18] | 1 | 0.0245 | * | * |  |  |
| K11635 | putative ABC transport system ATP-binding protein | 1 | 0.0208 |  | * |  |  |
| K11636 | putative ABC transport system permease protein | 0.992 | 0.0208 |  | * |  |  |
| K15760 | toluene monooxygenase system protein A [EC:1.14.13.236 1.14.13.-] | 1 | 0.0208 |  | * |  |  |
| K07705 | two-component system, LytTR family, response regulator LytT | 0.939 | 0.0486 | * | * |  |  |
| K07778 | two-component system, NarL family, sensor histidine kinase DesK [EC:2.7.13.3] | 0.993 | 0.0348 | * | * |  | * |
| K19623 | two-component system, probable response regulator PhcQ | 0.935 | 0.0385 |  | * |  |  |
| K19621 | two-component system, sensor histidine kinase PhcS [EC:2.7.13.3] | 1 | 0.0208 |  | * |  |  |
| K02117 | V/A-type H+/Na+-transporting ATPase subunit A [EC:3.6.3.14 3.6.3.15] | 0.999 | 0.0164 | * | * | * |  |
| K02118 | V/A-type H+/Na+-transporting ATPase subunit B | 1 | 0.0189 | * | * | * |  |
| K02120 | V/A-type H+/Na+-transporting ATPase subunit D | 1 | 0.0189 | * | * | * |  |
| K02121 | V/A-type H+/Na+-transporting ATPase subunit E | 0.999 | 0.0388 | * | * | * |  |
| K02122 | V/A-type H+/Na+-transporting ATPase subunit F | 1 | 0.0189 | * | * | * |  |
| K02124 | V/A-type H+/Na+-transporting ATPase subunit K | 0.997 | 0.0388 | * | * | * |  |
| K07260 | zinc D-Ala-D-Ala carboxypeptidase [EC:3.4.17.14] | 0.978 | 0.0171 | * | * |  | * |
| K12994 | mannosyl-N-acetyl-alpha-D-glucosaminyl-diphospho-ditrans,octacis-undecaprenol 3-alpha-mannosyltransferase / alpha-1,3-rhamnosyltransferase [EC:2.4.1.349 2.4.1.-] | 1 | 0.0057 | ** | ** |  |  |
| K13543 | uroporphyrinogen III methyltransferase / synthase [EC:2.1.1.107 4.2.1.75] | 1 | 0.0057 | ** | ** |  |  |
| K02123 | V/A-type H+/Na+-transporting ATPase subunit I | 0.999 | 0.0096 | ** | ** | ** |  |
| K18284 | adenosylhomocysteine/aminodeoxyfutalosine nucleosidase [EC:3.2.2.9 3.2.2.30] | 1 | 0.0211 | * |  |  |  |
| K05873 | adenylate cyclase, class 2 [EC:4.6.1.1] | 0.966 | 0.0351 | * |  | * |  |
| K00002 | alcohol dehydrogenase (NADP+) [EC:1.1.1.2] | 0.943 | 0.0382 |  |  | * |  |
| K11358 | aspartate aminotransferase [EC:2.6.1.1] | 1 | 0.0208 | * |  | * | * |
| K11782 | chorismate dehydratase [EC:4.2.1.151] | 0.997 | 0.0208 | * |  | * | * |
| K00442 | coenzyme F420 hydrogenase subunit delta | 1 | 0.0182 |  |  | * |  |
| K19091 | CRISPR-associated endoribonuclease Cas6 [EC:3.1.-.-] | 0.97 | 0.0242 |  |  | * |  |
| K07031 | D-glycero-alpha-D-manno-heptose-7-phosphate kinase [EC:2.7.1.168] | 0.974 | 0.0328 | * |  | * |  |
| K18967 | diguanylate cyclase [EC:2.7.7.65] | 0.974 | 0.0336 | * |  |  | * |
| K19171 | DNA sulfur modification protein DndD | 0.96 | 0.0189 |  |  |  | * |
| K14126 | F420-non-reducing hydrogenase large subunit [EC:1.12.99.- 1.8.98.5] | 1 | 0.0182 |  |  | * |  |
| K14128 | F420-non-reducing hydrogenase small subunit [EC:1.12.99.- 1.8.98.5] | 1 | 0.0182 |  |  | * |  |
| K11261 | formylmethanofuran dehydrogenase subunit E [EC:1.2.7.12] | 0.999 | 0.0409 | * |  | * | * |
| K19510 | fructoselysine-6-phosphate deglycase | 1 | 0.0057 |  |  | ** | ** |
| K11017 | hemolysin activation/secretion protein | 0.981 | 0.0387 |  |  |  | * |
| K03388 | heterodisulfide reductase subunit A2 [EC:1.8.7.3 1.8.98.4 1.8.98.5 1.8.98.6] | 1 | 0.0208 | * |  | * | * |
| K03390 | heterodisulfide reductase subunit C2 [EC:1.8.7.3 1.8.98.4 1.8.98.5 1.8.98.6] | 1 | 0.0208 | * |  | * | * |
| K04486 | histidinol-phosphatase (PHP family) [EC:3.1.3.15] | 0.997 | 0.0064 | ** |  | ** | ** |
| K00179 | indolepyruvate ferredoxin oxidoreductase, alpha subunit [EC:1.2.7.8] | 0.995 | 0.0376 | * |  | * | * |
| K00180 | indolepyruvate ferredoxin oxidoreductase, beta subunit [EC:1.2.7.8] | 0.997 | 0.0428 | * |  | * | * |
| K10206 | LL-diaminopimelate aminotransferase [EC:2.6.1.83] | 0.998 | 0.0208 | * |  | * | * |
| K06864 | pyridinium-3,5-biscarboxylic acid mononucleotide sulfurtransferase | 0.994 | 0.0208 | * |  | * | * |
| K00171 | pyruvate ferredoxin oxidoreductase delta subunit [EC:1.2.7.1] | 1 | 0.0211 | * |  |  |  |
| K06381 | stage II sporulation protein D | 0.999 | 0.021 | * |  | * |  |
| K15372 | taurine---2-oxoglutarate transaminase [EC:2.6.1.55] | 0.981 | 0.0133 | * |  |  | * |
| K13770 | TetR/AcrR family transcriptional regulator, fatty acid metabolism regulator protein | 0.997 | 0.0208 | * |  | * | * |
| K04720 | threonine-phosphate decarboxylase [EC:4.1.1.81] | 0.999 | 0.0197 | * |  | * |  |
| K02482 | two-component system, NtrC family, sensor kinase [EC:2.7.13.3] | 0.988 | 0.0399 | * |  | * | * |
| K01163 | uncharacterized protein | 0.996 | 0.0148 | * |  | * |  |
| K05967 | uncharacterized protein | 0.997 | 0.0208 | * |  | * | * |
| K09740 | uncharacterized protein | 1 | 0.0208 | * |  | * | * |
| K09777 | uncharacterized protein | 0.999 | 0.0208 | * |  | * | * |
| K09707 | uncharacterized protein | 1 | 0.0211 | * |  |  |  |
| K02119 | V/A-type H+/Na+-transporting ATPase subunit C | 0.98 | 0.0241 | * |  | * |  |
| **Epimicrobiome** | | | | | | | |
| **Day 3 - directly after stress** | | | | | | | |
| K14470 | 2-methylfumaryl-CoA isomerase [EC:5.4.1.3] | 0.927 | 0.0337 |  | * | * | * |
| K05797 | 4-cresol dehydrogenase (hydroxylating) flavoprotein subunit [EC:1.17.99.1] | 0.812 | 0.0428 |  | * | * |  |
| K10211 | 4,4'-diaponeurosporenoate glycosyltransferase [EC:2.4.1.-] | 0.902 | 0.0219 |  | * | * | * |
| K12269 | accessory secretory protein Asp2 | 0.758 | 0.0482 |  | * | * |  |
| K12292 | ATP-binding cassette, subfamily C, bacterial, competence factor transporting protein [EC:3.4.22.-] | 0.764 | 0.0239 |  | * | * |  |
| K10910 | autoinducer 2-binding periplasmic protein LuxP | 0.975 | 0.0405 |  | * | * | * |
| K01060 | cephalosporin-C deacetylase [EC:3.1.1.41] | 0.84 | 0.0423 | * | * | * |  |
| K03333 | cholesterol oxidase [EC:1.1.3.6] | 0.958 | 0.0491 | * | * | * |  |
| K19073 | divinyl chlorophyllide a 8-vinyl-reductase [EC:1.3.1.75] | 0.924 | 0.0183 |  | * | * | * |
| K13797 | DNA-directed RNA polymerase subunit beta-beta' [EC:2.7.7.6] | 0.707 | 0.0388 |  | * |  |  |
| K16559 | endo-1,3-1,4-beta-glycanase ExoK [EC:3.2.1.-] | 0.859 | 0.0198 |  | * |  |  |
| K02639 | ferredoxin | 0.906 | 0.0335 |  | * | * | * |
| K00118 | glucose-fructose oxidoreductase [EC:1.1.99.28] | 0.911 | 0.0273 |  | * | * | * |
| K00693 | glycogen synthase [EC:2.4.1.11] | 0.779 | 0.0294 |  | * |  |  |
| K10212 | glycosyl-4,4'-diaponeurosporenoate acyltransferase [EC:2.3.1.-] | 0.895 | 0.0298 |  | * | * | * |
| K07215 | heme oxygenase (biliverdin-IX-beta and delta-forming) [EC:1.14.99.58] | 0.978 | 0.0245 | * | * | * |  |
| K19350 | lincosamide and streptogramin A transport system ATP-binding/permease protein | 0.841 | 0.0155 |  | * |  | * |
| K08692 | malate-CoA ligase subunit alpha [EC:6.2.1.9] | 0.903 | 0.0294 |  | * | * | * |
| K14067 | malate-CoA ligase subunit beta [EC:6.2.1.9] | 0.9 | 0.0362 |  | * | * | * |
| K00217 | maleylacetate reductase [EC:1.3.1.32] | 0.664 | 0.0466 |  | * |  |  |
| K09819 | manganese/iron transport system permease protein | 0.94 | 0.0152 |  | * | * | * |
| K01461 | N-acyl-D-glutamate deacylase [EC:3.5.1.82] | 0.906 | 0.0415 |  | * | * | * |
| K01003 | oxaloacetate decarboxylase [EC:4.1.1.3] | 0.949 | 0.0332 | * | * | * |  |
| K09459 | phosphonopyruvate decarboxylase [EC:4.1.1.82] | 0.754 | 0.0455 |  | * | * |  |
| K18067 | phthalate 4,5-cis-dihydrodiol dehydrogenase [EC:1.3.1.64] | 0.903 | 0.0188 |  | * | * | * |
| K19697 | propionate kinase [EC:2.7.2.15] | 0.707 | 0.0353 |  | * |  |  |
| K04712 | sphingolipid 4-desaturase/C4-monooxygenase [EC:1.14.19.17 1.14.18.5] | 0.799 | 0.0437 |  | * |  | * |
| K06045 | squalene-hopene/tetraprenyl-beta-curcumene cyclase [EC:5.4.99.17 4.2.1.129] | 0.847 | 0.0423 |  | * |  | * |
| K16562 | succinoglycan biosynthesis protein ExoW [EC:2.4.-.-] | 0.785 | 0.0184 |  | * |  |  |
| K00917 | tagatose 6-phosphate kinase [EC:2.7.1.144] | 0.902 | 0.0112 |  | * | * | * |
| K19519 | transforming growth factor-beta-induced protein | 0.764 | 0.0366 |  | * | * |  |
| K19661 | two-component system, NtrC family, sensor histidine kinase HupT/HoxJ [EC:2.7.13.3] | 0.874 | 0.0144 |  | * | * | * |
| K11898 | type VI secretion system protein ImpE | 0.955 | 0.0375 |  | * | * | * |
| K08068 | UDP-N-acetylglucosamine 2-epimerase (hydrolysing) [EC:3.2.1.183] | 0.923 | 0.0297 |  | * | * | * |
| K09155 | uncharacterized protein | 0.938 | 0.0137 |  | * | * |  |
| K09144 | uncharacterized protein | 0.832 | 0.0223 |  | * |  |  |
| K00996 | undecaprenyl-phosphate galactose phosphotransferase [EC:2.7.8.6] | 0.904 | 0.0352 | * | * | * |  |
| K01198 | xylan 1,4-beta-xylosidase [EC:3.2.1.37] | 0.94 | 0.0465 |  | * | * | * |
| K07260 | zinc D-Ala-D-Ala carboxypeptidase [EC:3.4.17.14] | 0.966 | 0.0341 | * | * | * |  |
| K16927 | energy-coupling factor transport system substrate-specific component | 0.957 | 0.0044 |  | ** | ** | ** |
| K16190 | glucuronokinase [EC:2.7.1.43] | 0.816 | 0.0063 |  | ** |  |  |
| K00613 | glycine amidinotransferase [EC:2.1.4.1] | 0.961 | 0.0048 |  | ** | ** | ** |
| K16555 | succinoglycan biosynthesis protein ExoO [EC:2.4.-.-] | 0.93 | 0.0047 |  | ** |  |  |
| K06873 | uncharacterized protein | 0.941 | 0.007 |  | ** | ** |  |
| K14956 | 6 kDa early secretory antigenic target | 0.775 | 0.0137 | * |  |  |  |
| K00842 | aminotransferase [EC:2.6.1.-] | 0.721 | 0.0286 | * |  |  |  |
| K18701 | arsenate-mycothiol transferase [EC:2.8.4.2] | 0.743 | 0.0333 | * |  |  |  |
| K14698 | ATP-binding cassette, subfamily B, bacterial IrtA [EC:3.6.3.-] | 0.741 | 0.0286 | * |  |  |  |
| K14699 | ATP-binding cassette, subfamily B, bacterial IrtB [EC:3.6.3.-] | 0.759 | 0.0141 | * |  |  |  |
| K18895 | ATP-binding cassette, subfamily B, salmochelin/enterobactin exporter | 0.705 | 0.0296 | * |  |  |  |
| K11953 | bicarbonate transport system ATP-binding protein [EC:3.6.3.-] | 0.769 | 0.0305 |  |  |  | * |
| K13381 | bifunctional chitinase/lysozyme [EC:3.2.1.14 3.2.1.17] | 0.929 | 0.0322 | * |  | * | * |
| K07291 | CDP-L-myo-inositol myo-inositolphosphotransferase [EC:2.7.8.34] | 0.789 | 0.0242 |  |  | * |  |
| K18589 | dihydrofolate reductase (trimethoprim resistance protein) [EC:1.5.1.3] | 0.775 | 0.0122 | * |  |  |  |
| K18288 | itaconate CoA-transferase [EC:2.8.3.-] | 0.817 | 0.0183 |  |  | * |  |
| K10215 | monooxygenase [EC:1.14.13.-] | 0.775 | 0.0137 | * |  |  |  |
| K13380 | NADH-quinone oxidoreductase subunit B/C/D [EC:1.6.5.3] | 0.819 | 0.0307 |  |  | * |  |
| K12227 | TraL protein | 0.775 | 0.0122 | * |  |  |  |
| **Day 12 - after the recovery period** | |  |  |  |  |  |  |
| K10216 | 2-hydroxymuconate-semialdehyde hydrolase [EC:3.7.1.9] | 0.945 | 0.0193 |  | * | * |  |
| K06968 | 23S rRNA (cytidine2498-2'-O)-methyltransferase [EC:2.1.1.186] | 1 | 0.0363 | * | * |  | * |
| K18013 | 3-keto-5-aminohexanoate cleavage enzyme [EC:2.3.1.247] | 0.955 | 0.0125 |  | * | * |  |
| K05296 | 3(or 17)beta-hydroxysteroid dehydrogenase [EC:1.1.1.51] | 0.994 | 0.0345 | * | * |  | * |
| K16020 | 5-deoxy-5-amino-3-dehydroquinate synthase | 0.998 | 0.0185 |  | * | * | * |
| K11744 | AI-2 transport protein TqsA | 0.998 | 0.0286 | * | * |  | * |
| K04022 | alcohol dehydrogenase | 1 | 0.0284 | * | * |  | * |
| K09023 | aminoacrylate hydrolase [EC:3.5.1.-] | 0.998 | 0.0393 | * | * |  | * |
| K03395 | aminoglycoside 3-N-acetyltransferase I [EC:2.3.1.60] | 0.955 | 0.0189 |  | * |  |  |
| K13631 | AraC family transcriptional regulator, mar-sox-rob regulon activator | 1 | 0.0299 | * | * |  | * |
| K13633 | AraC family transcriptional regulator, transcriptional activator FtrA | 0.991 | 0.0341 |  | * | * | * |
| K10000 | arginine transport system ATP-binding protein [EC:3.6.3.-] | 1 | 0.0193 | * | * |  | * |
| K14165 | atypical dual specificity phosphatase [EC:3.1.3.16 3.1.3.48] | 0.99 | 0.0276 | * | * |  | * |
| K01860 | chloromuconate cycloisomerase [EC:5.5.1.7] | 0.986 | 0.0189 |  | * |  |  |
| K15861 | CRP/FNR family transcriptional regulator, nitrogen fixation regulation protein | 0.924 | 0.0329 |  | * | * |  |
| K17899 | D-ornithine 4,5-aminomutase subunit alpha [EC:5.4.3.5] | 0.959 | 0.0189 |  | * | * |  |
| K17898 | D-ornithine 4,5-aminomutase subunit beta [EC:5.4.3.5] | 0.974 | 0.0189 |  | * | * |  |
| K03573 | DNA mismatch repair protein MutH | 1 | 0.0241 | * | * |  | * |
| K16923 | energy-coupling factor transport system substrate-specific component | 1 | 0.0193 | * | * |  | * |
| K04025 | ethanolamine utilization protein EutK | 1 | 0.0378 | * | * |  | * |
| K11600 | exosome complex component RRP41 | 0.952 | 0.0189 |  | * |  |  |
| K09473 | gamma-glutamyl-gamma-aminobutyrate hydrolase [EC:3.5.1.94] | 1 | 0.0193 | * | * |  | * |
| K00113 | glycerol-3-phosphate dehydrogenase subunit C [EC:1.1.5.3] | 0.996 | 0.0276 | * | * |  | * |
| K10670 | glycine/sarcosine/betaine reductase complex component A [EC:1.21.4.2 1.21.4.3 1.21.4.4] | 0.962 | 0.0189 |  | * | * |  |
| K05839 | haemolysin expression modulating protein | 1 | 0.0193 | * | * |  | * |
| K02848 | heptose I phosphotransferase [EC:2.7.1.-] | 1 | 0.0363 | * | * |  | * |
| K13779 | isohexenylglutaconyl-CoA hydratase [EC:4.2.1.57] | 1 | 0.0193 | * | * |  | * |
| K00103 | L-gulonolactone oxidase [EC:1.1.3.8] | 0.996 | 0.0185 |  | * | * | * |
| K14733 | limonene 1,2-monooxygenase [EC:1.14.13.107] | 1 | 0.0185 |  | * | * | * |
| K18231 | macrolide transport system ATP-binding/permease protein | 0.907 | 0.0483 |  | * | * |  |
| K15974 | MarR family transcriptional regulator, negative regulator of the multidrug operon emrRAB | 1 | 0.0193 | * | * |  | * |
| K05375 | MbtH protein | 0.994 | 0.0481 | * | * |  | * |
| K13638 | MerR family transcriptional regulator, Zn(II)-responsive regulator of zntA | 1 | 0.0373 | * | * |  | * |
| K08193 | MFS transporter, ACS family, solute carrier family 17 (sodium-dependent inorganic phosphate cotransporter), other | 0.946 | 0.0189 |  | * |  |  |
| K03445 | MFS transporter, DHA1 family, purine ribonucleoside efflux pump | 0.999 | 0.0151 | * | * |  | * |
| K01787 | N-acylglucosamine 2-epimerase [EC:5.1.3.8] | 0.992 | 0.0341 |  | * | * | * |
| K01459 | N-carbamoyl-D-amino-acid hydrolase [EC:3.5.1.77] | 0.993 | 0.0185 |  | * | * | * |
| K16243 | Phenol hydroxylase P1 protein | 0.918 | 0.0394 |  | * | * |  |
| K01002 | phosphoglycerol transferase [EC:2.7.8.20] | 1 | 0.0363 | * | * |  | * |
| K16328 | pseudouridine kinase [EC:2.7.1.83] | 0.999 | 0.0283 | * | * |  | * |
| K02775 | PTS system, galactitol-specific IIC component | 1 | 0.0404 | * | * |  | * |
| K08776 | puromycin-sensitive aminopeptidase [EC:3.4.11.-] | 0.998 | 0.0129 |  | * | * | * |
| K19430 | pyridoxal phosphate-dependent aminotransferase EpsN [EC:2.6.1.-] | 0.951 | 0.0257 |  | * | * |  |
| K09893 | regulator of ribonuclease activity B | 1 | 0.0393 | * | * |  | * |
| K18148 | release factor H-coupled RctB family protein | 0.999 | 0.0382 | * | * |  | * |
| K08324 | succinate-semialdehyde dehydrogenase [EC:1.2.1.16 1.2.1.24] | 1 | 0.0404 | * | * |  | * |
| K15064 | syringate O-demethylase [EC:2.1.1.-] | 0.949 | 0.0189 |  | * |  |  |
| K03838 | threonine transporter | 1 | 0.0193 | * | * |  | * |
| K15765 | toluene monooxygenase electron transfer component [EC:1.18.1.3] | 0.97 | 0.0369 |  | * |  |  |
| K15764 | toluene monooxygenase system protein E [EC:1.14.13.236 1.14.13.-] | 0.959 | 0.0189 |  | * |  |  |
| K19155 | toxin YhaV [EC:3.1.-.-] | 0.999 | 0.036 | * | * |  | * |
| K11624 | two-component system, NarL family, response regulator YdfI | 0.953 | 0.0189 |  | * |  |  |
| K09957 | uncharacterized protein | 0.99 | 0.017 |  | * | * | * |
| K09128 | uncharacterized protein | 0.947 | 0.0189 |  | * |  |  |
| K07070 | uncharacterized protein | 1 | 0.0193 | * | * |  | * |
| K09907 | uncharacterized protein | 1 | 0.0197 | * | * |  | * |
| K09974 | uncharacterized protein | 0.996 | 0.0372 | * | * |  | * |
| K08997 | uncharacterized protein | 1 | 0.0413 | * | * |  | * |
| K00365 | urate oxidase [EC:1.7.3.3] | 0.967 | 0.0259 |  | * | * |  |
| K00073 | ureidoglycolate dehydrogenase (NAD+) [EC:1.1.1.350] | 0.999 | 0.0382 | * | * |  | * |
| K15066 | vanillate/3-O-methylgallate O-demethylase [EC:2.1.1.341] | 0.913 | 0.0469 |  | * |  | * |
| K05881 | phosphoenolpyruvate---glycerone phosphotransferase subunit DhaM [EC:2.7.1.121] | 1 | 0.0081 | ** | ** |  | ** |
| K09916 | uncharacterized protein | 0.999 | 0.0072 | ** | ** |  | ** |
| K16840 | 2-oxo-4-hydroxy-4-carboxy-5-ureidoimidazoline decarboxylase [EC:4.1.1.97] | 0.992 | 0.0067 | ** |  |  | ** |
| K03078 | 3-dehydro-L-gulonate-6-phosphate decarboxylase [EC:4.1.1.85] | 0.966 | 0.0403 | * |  |  | * |
| K05709 | 3-phenylpropionate/trans-cinnamate dioxygenase subunit beta [EC:1.14.12.19] | 0.959 | 0.0403 | * |  |  | * |
| K12507 | acyl-CoA synthetase [EC:6.2.1.-] | 0.957 | 0.0454 | * |  |  | * |
| K11739 | bacteriophage N4 adsorption protein A | 0.963 | 0.0323 | * |  |  | * |
| K00141 | benzaldehyde dehydrogenase (NAD) [EC:1.2.1.28] | 0.97 | 0.0266 | * |  |  | * |
| K06197 | cation transport regulator | 0.956 | 0.0198 | * |  |  | * |
| K18657 | cell division protein ZapC | 0.967 | 0.0255 | * |  |  | * |
| K12055 | chromosome partitioning related protein ParA | 0.955 | 0.0354 | * |  |  | * |
| K09477 | citrate:succinate antiporter | 0.958 | 0.0337 | * |  |  | * |
| K11249 | cysteine/O-acetylserine efflux protein | 0.957 | 0.0331 | * |  |  | * |
| K18968 | diguanylate cyclase [EC:2.7.7.65] | 0.957 | 0.0241 | * |  |  | * |
| K12961 | DnaA initiator-associating protein | 0.966 | 0.0331 | * |  |  | * |
| K08276 | ecotin | 0.958 | 0.0435 | * |  |  | * |
| K00435 | Fe-coproporphyrin III decarboxylase [EC:1.11.1.-] | 0.997 | 0.0344 | * |  |  | * |
| K05997 | Fe-S cluster assembly protein SufA | 0.956 | 0.0384 | * |  |  | * |
| K15836 | formate hydrogenlyase transcriptional activator | 0.956 | 0.0337 | * |  |  | * |
| K10708 | fructoselysine 6-phosphate deglycase [EC:3.5.-.-] | 0.962 | 0.0497 | * |  |  | * |
| K19540 | fructoselysine transporter | 0.963 | 0.0403 | * |  |  | * |
| K11735 | GABA permease | 0.964 | 0.0475 | * |  |  | * |
| K10036 | glutamine transport system substrate-binding protein | 0.959 | 0.05 | * |  |  | * |
| K03674 | glutaredoxin 1 | 0.968 | 0.0403 | * |  |  | * |
| K19775 | GntR family transcriptional regulator, hexuronate regulon transcriptional repressor | 0.959 | 0.0198 | * |  |  | * |
| K16152 | heme acquisition protein HasR | 0.945 | 0.0453 | * |  |  | * |
| K11139 | hemolysin E | 0.957 | 0.0493 | * |  |  | * |
| K02850 | heptose II phosphotransferase [EC:2.7.1.-] | 0.957 | 0.0447 | * |  |  | * |
| K19354 | heptose III glucuronosyltransferase [EC:2.4.1.-] | 0.957 | 0.0403 | * |  |  | * |
| K03382 | hydroxydechloroatrazine ethylaminohydrolase [EC:3.5.4.43] | 0.913 | 0.0438 |  |  | * | * |
| K10530 | L-lactate oxidase [EC:1.1.3.2] | 0.977 | 0.0364 |  |  |  | * |
| K19236 | L,D-transpeptidase YcfS | 0.961 | 0.0337 | * |  |  | * |
| K05803 | lipoprotein NlpI | 0.966 | 0.0331 | * |  |  | * |
| K08159 | MFS transporter, DHA1 family, L-arabinose/isopropyl-beta-D-thiogalactopyranoside export protein | 0.955 | 0.0472 | * |  |  | * |
| K07349 | minor fimbrial subunit | 0.958 | 0.0264 | * |  |  | * |
| K19157 | mRNA interferase YafQ [EC:3.1.-.-] | 0.933 | 0.0391 | * |  |  | * |
| K06159 | multidrug/microcin transport system ATP-binding/permease protein | 0.954 | 0.0481 | * |  |  | * |
| K10201 | N-acetylglucosamine transport system permease protein | 1 | 0.0185 | * |  |  |  |
| K06006 | periplasmic protein CpxP | 0.966 | 0.0377 | * |  |  | * |
| K02507 | protein transport protein HofQ | 0.961 | 0.023 | * |  |  | * |
| K02815 | PTS system, sorbose-specific IID component | 0.965 | 0.016 | * |  |  | * |
| K09016 | putative pyrimidine permease RutG | 0.96 | 0.0383 | * |  |  | * |
| K05358 | quinate dehydrogenase (quinone) [EC:1.1.5.8] | 0.959 | 0.0454 | * |  |  | * |
| K05809 | ribosome-associated inhibitor A | 0.964 | 0.0337 | * |  |  | * |
| K15723 | SecY interacting protein Syd | 0.969 | 0.0307 | * |  |  | * |
| K00840 | succinylornithine aminotransferase [EC:2.6.1.81] | 0.956 | 0.0383 | * |  |  | * |
| K08357 | tetrathionate reductase subunit A | 0.963 | 0.0375 | * |  |  |  |
| K08359 | tetrathionate reductase subunit C | 0.968 | 0.0375 | * |  |  |  |
| K18446 | triphosphatase [EC:3.6.1.25] | 0.965 | 0.0319 | * |  |  | * |
| K09917 | uncharacterized protein | 0.961 | 0.0198 | * |  |  | * |
| K09934 | uncharacterized protein | 0.953 | 0.0337 | * |  |  | * |
| K11932 | universal stress protein G | 0.965 | 0.0403 | * |  |  | * |
| K06074 | vitamin B12 transport system ATP-binding protein [EC:3.6.3.33] | 0.961 | 0.0383 | * |  |  | * |
| K06073 | vitamin B12 transport system permease protein | 0.966 | 0.0264 | * |  |  | * |
| **Rhizomicrobiome** | | | | | | | |
| **Day 3 - directly after stress** | | | | | | | |
| K00596 | 2,2-dialkylglycine decarboxylase (pyruvate) [EC:4.1.1.64] | 0.949 | 0.0132 | * | * |  |  |
| K05281 | 2'-hydroxyisoflavone reductase [EC:1.3.1.45] | 0.981 | 0.0259 | * | * | * |  |
| K18013 | 3-keto-5-aminohexanoate cleavage enzyme [EC:2.3.1.247] | 0.995 | 0.0338 | * | * |  | * |
| K00082 | 5-amino-6-(5-phosphoribosylamino)uracil reductase [EC:1.1.1.193] | 0.988 | 0.0189 | * | * |  | * |
| K06714 | arginine utilization regulatory protein | 0.987 | 0.048 | * | * |  | * |
| K01844 | beta-lysine 5,6-aminomutase alpha subunit [EC:5.4.3.3] | 0.998 | 0.0363 | * | * |  | * |
| K17899 | D-ornithine 4,5-aminomutase subunit alpha [EC:5.4.3.5] | 0.992 | 0.0418 | * | * |  | * |
| K17898 | D-ornithine 4,5-aminomutase subunit beta [EC:5.4.3.5] | 0.994 | 0.0341 | * | * |  | * |
| K13444 | formylglycine-generating enzyme [EC:1.8.3.7] | 0.913 | 0.0296 |  | * |  | * |
| K10670 | glycine/sarcosine/betaine reductase complex component A [EC:1.21.4.2 1.21.4.3 1.21.4.4] | 0.998 | 0.0181 | * | * |  | * |
| K13927 | holo-ACP synthase / triphosphoribosyl-dephospho-CoA synthase [EC:2.7.7.61 2.4.2.52] | 0.906 | 0.0304 |  | * |  | * |
| K11051 | multidrug/hemolysin transport system permease protein | 0.967 | 0.0131 |  | * |  | * |
| K18068 | phthalate 4,5-dioxygenase [EC:1.14.12.7] | 0.913 | 0.0337 |  | * |  | * |
| K08252 | receptor protein-tyrosine kinase [EC:2.7.10.1] | 0.943 | 0.0221 | * | * |  | * |
| K01822 | steroid Delta-isomerase [EC:5.3.3.1] | 0.982 | 0.0159 | * | * |  | * |
| K01502 | aliphatic nitrilase [EC:3.5.5.7] | 0.985 | 0.0057 | ** | ** |  |  |
| K01014 | aryl sulfotransferase [EC:2.8.2.1] | 0.963 | 0.0065 |  | ** |  | ** |
| K01045 | arylesterase / paraoxonase [EC:3.1.1.2 3.1.8.1] | 1 | 0.0019 | ** | ** |  | ** |
| K18481 | Mce-associated membrane protein | 0.957 | 0.0082 |  | ** |  | ** |
| K10555 | AI-2 transport system substrate-binding protein | 0.987 | 0.0194 | * |  | * | * |
| K12206 | intracellular multiplication protein IcmB | 0.907 | 0.0479 | * |  | * |  |
| K12217 | intracellular multiplication protein IcmO | 0.915 | 0.0409 | * |  |  |  |
| K12222 | intracellular multiplication protein IcmT | 0.961 | 0.0231 | * |  |  |  |
| K13001 | mannosyltransferase [EC:2.4.1.-] | 1 | 0.0124 | * |  |  |  |
| K01730 | oligogalacturonide lyase [EC:4.2.2.6] | 0.947 | 0.0049 | ** |  |  |  |
| K01569 | oxalate decarboxylase [EC:4.1.1.2] | 0.968 | 0.0026 | ** |  |  |  |
| K19640 | putative two-component system protein, hydrogenase maturation factor HypX/HoxX | 0.959 | 0.0068 | ** |  |  | ** |
| K10213 | ribosylpyrimidine nucleosidase [EC:3.2.2.8] | 0.977 | 0.0041 | ** |  | ** | ** |
| K02107 | V/A-type H+/Na+-transporting ATPase subunit G/H | 0.953 | 0.0431 | * |  | * |  |
| **Day 12 - after the recovery period** | | | | | | | |
| K08321 | 3-hydroxy-5-phosphonooxypentane-2,4-dione thiolase [EC:2.3.1.245] | 0.97 | 0.0394 |  | * | * | * |
| K16051 | 3-oxo-5alpha-steroid 4-dehydrogenase [EC:1.3.99.5] | 0.968 | 0.0351 |  | * |  | * |
| K16050 | 4,5:9,10-diseco-3-hydroxy-5,9,17-trioxoandrosta-1(10),2-diene-4-oate hydrolase [EC:3.7.1.17] | 0.99 | 0.05 | * | * |  | * |
| K19572 | adenosine deaminase CECR1 [EC:3.5.4.4] | 0.993 | 0.013 |  | * | * | * |
| K02099 | AraC family transcriptional regulator, arabinose operon regulatory protein | 0.973 | 0.0271 |  | * | * | * |
| K19115 | CRISPR-associated protein Csh2 | 0.975 | 0.031 | * | * | * |  |
| K03146 | cysteine-dependent adenosine diphosphate thiazole synthase [EC:2.4.2.60] | 0.866 | 0.0323 |  | * |  |  |
| K00007 | D-arabinitol 4-dehydrogenase [EC:1.1.1.11] | 0.934 | 0.0302 |  | * | * | * |
| K08349 | formate dehydrogenase-N, beta subunit | 0.987 | 0.0132 |  | * | * | * |
| K04018 | formate-dependent nitrite reductase complex subunit NrfG | 0.982 | 0.0288 |  | * | * | * |
| K04103 | indolepyruvate decarboxylase [EC:4.1.1.74] | 0.96 | 0.0151 |  | * | * | * |
| K03079 | L-ribulose-5-phosphate 3-epimerase [EC:5.1.3.22] | 0.984 | 0.0417 |  | * | * | * |
| K19235 | L,D-transpeptidase YbiS | 0.973 | 0.0445 |  | * | * | * |
| K12113 | LacI family transcriptional regulator, ebg operon repressor | 0.983 | 0.0471 |  | * | * | * |
| K12978 | lipid A 4'-phosphatase [EC:3.1.3.-] | 0.933 | 0.0263 |  | * | * |  |
| K02562 | mannitol operon repressor | 0.988 | 0.0294 |  | * | * | * |
| K18141 | membrane fusion protein, multidrug efflux system | 0.975 | 0.022 |  | * | * | * |
| K08154 | MFS transporter, DHA1 family, 2-module integral membrane pump EmrD | 0.975 | 0.015 |  | * | * | * |
| K13650 | MqsR-controlled colanic acid and biofilm protein A | 0.953 | 0.0258 |  | * | * | * |
| K01137 | N-acetylglucosamine-6-sulfatase [EC:3.1.6.14] | 0.88 | 0.0213 |  | * | * |  |
| K10678 | nitroreductase [EC:1.-.-.-] | 0.981 | 0.0348 |  | * | * | * |
| K16249 | phenol hydroxylase P0 protein | 0.987 | 0.0361 |  | * | * | * |
| K16243 | Phenol hydroxylase P1 protein | 0.987 | 0.0276 |  | * | * | * |
| K01096 | phosphatidylglycerophosphatase B [EC:3.1.3.27 3.1.3.81 3.1.3.4 3.6.1.27] | 0.981 | 0.0426 |  | * | * | * |
| K12289 | pilus assembly protein HofN | 0.98 | 0.0495 |  | * | * | * |
| K02822 | PTS system, ascorbate-specific IIB component [EC:2.7.1.194] | 0.989 | 0.0454 |  | * | * | * |
| K18531 | putative frv operon regulatory protein | 0.973 | 0.0184 |  | * | * | * |
| K10762 | putative replication protein | 0.953 | 0.0296 |  | * | * | * |
| K03093 | RNA polymerase sigma factor | 0.978 | 0.0288 | * | * | * |  |
| K18988 | serine-type D-Ala-D-Ala carboxypeptidase/endopeptidase [EC:3.4.16.4 3.4.21.-] | 0.983 | 0.0123 |  | * | * | * |
| K07309 | Tat-targeted selenate reductase subunit YnfE [EC:1.97.1.9] | 0.987 | 0.036 |  | * | * | * |
| K15765 | toluene monooxygenase electron transfer component [EC:1.18.1.3] | 0.889 | 0.0198 |  | * |  | * |
| K07647 | two-component system, OmpR family, sensor histidine kinase TorS [EC:2.7.13.3] | 0.977 | 0.036 |  | * | * | * |
| K07149 | uncharacterized protein | 0.963 | 0.0203 |  | * | * | * |
| K17251 | undecaprenyl-diphosphooligosaccharide---protein glycotransferase [EC:2.4.99.19] | 0.984 | 0.0313 | * | * | * |  |
| K18581 | unsaturated chondroitin disaccharide hydrolase [EC:3.2.1.180] | 0.974 | 0.0425 |  | * | * | * |
| K19156 | antitoxin PrlF | 0.994 | 0.0094 |  | ** | ** | ** |
| K15750 | biphenyl 2,3-dioxygenase subunit beta [EC:1.14.12.18] | 0.958 | 0.0042 |  | ** | ** |  |
| K01565 | N-sulfoglucosamine sulfohydrolase [EC:3.10.1.1] | 0.971 | 0.0074 |  | ** |  |  |
| K04101 | protocatechuate 4,5-dioxygenase, beta chain [EC:1.13.11.8] | 0.998 | 0.0012 |  | ** | ** | ** |
| K15024 | putative phosphotransacetylase [EC:2.3.1.8] | 0.995 | 0.0028 |  | ** | ** | ** |
| K11031 | thiol-activated cytolysin | 0.943 | 0.0058 |  | ** | ** | ** |
| K10219 | 2-hydroxy-4-carboxymuconate semialdehyde hemiacetal dehydrogenase [EC:1.1.1.312] | 0.952 | 0.0428 |  |  | * | * |
| K00561 | 23S rRNA (adenine-N6)-dimethyltransferase [EC:2.1.1.184] | 0.873 | 0.0488 |  |  |  | * |
| K13669 | alpha-1,2-mannosyltransferase [EC:2.4.1.-] | 0.775 | 0.0357 | * |  |  |  |
| K12940 | aminobenzoyl-glutamate utilization protein A | 0.951 | 0.033 |  |  | * | * |
| K16647 | arabinofuranan 3-O-arabinosyltransferase [EC:2.4.2.47] | 0.89 | 0.0431 |  |  | * | * |
| K13687 | arabinofuranosyltransferase [EC:2.4.2.-] | 0.972 | 0.0127 |  |  | * | * |
| K11953 | bicarbonate transport system ATP-binding protein [EC:3.6.3.-] | 0.898 | 0.0442 |  |  |  | * |
| K19688 | biofilm regulator BssR | 0.946 | 0.0435 | * |  | * | * |
| K12954 | cation-transporting P-type ATPase G [EC:3.6.3.-] | 0.904 | 0.0468 |  |  | * | * |
| K16269 | cis-1,2-dihydrobenzene-1,2-diol dehydrogenase [EC:1.3.1.19 1.3.1.-] | 0.881 | 0.0449 |  |  | * |  |
| K08690 | cis-2,3-dihydrobiphenyl-2,3-diol dehydrogenase [EC:1.3.1.56] | 0.879 | 0.0315 |  |  | * |  |
| K09002 | CRISPR-associated protein Csm3 | 0.836 | 0.0498 |  |  | * |  |
| K10550 | D-allose transport system permease protein | 0.87 | 0.0259 |  |  |  | * |
| K15733 | dye decolorizing peroxidase [EC:1.11.1.19] | 0.984 | 0.0203 |  |  | * | * |
| K07351 | fimbrial protein | 0.945 | 0.0397 |  |  | * | * |
| K04099 | gallate dioxygenase [EC:1.13.11.57] | 0.775 | 0.0342 | * |  |  |  |
| K06211 | HTH-type transcriptional regulator, transcriptional repressor of NAD biosynthesis genes [EC:2.7.7.1 2.7.1.22] | 0.979 | 0.012 | * |  | * | * |
| K12139 | hydrogenase-4 component D [EC:1.-.-.-] | 0.938 | 0.0116 |  |  | * | * |
| K12145 | hydrogenase-4 component J [EC:1.-.-.-] | 0.992 | 0.0018 | ** |  | ** | ** |
| K18541 | mandelamide amidase [EC:3.5.1.86] | 0.934 | 0.0396 |  |  | * |  |
| K18833 | MFS transporter, DHA3 family, multidrug efflux protein | 0.811 | 0.0489 |  |  |  | * |
| K11537 | MFS transporter, NHS family, xanthosine permease | 0.911 | 0.0358 |  |  |  | * |
| K10215 | monooxygenase [EC:1.14.13.-] | 0.913 | 0.0471 |  |  | * | * |
| K16870 | N-acetylglucosaminyl-diphospho-decaprenol L-rhamnosyltransferase [EC:2.4.1.289] | 0.961 | 0.0192 |  |  | * | * |
| K03418 | N,N-dimethylformamidase [EC:3.5.1.56] | 0.962 | 0.0058 |  |  | ** | ** |
| K12349 | neutral ceramidase [EC:3.5.1.23] | 0.95 | 0.0435 |  |  |  | * |
| K10018 | octopine/nopaline transport system substrate-binding protein | 0.977 | 0.0126 |  |  | * | * |
| K08724 | penicillin-binding protein 2B | 0.983 | 0.0433 | * |  | * | * |
| K02812 | PTS system, sorbose-specific IIA component [EC:2.7.1.206] | 0.965 | 0.0169 |  |  | * | * |
| K18148 | release factor H-coupled RctB family protein | 0.988 | 0.0139 |  |  | * | * |
| K05886 | serine 3-dehydrogenase (NADP+) [EC:1.1.1.276] | 0.991 | 0.0038 |  |  |  | ** |
| K06306 | spore germination protein | 0.957 | 0.0175 | * |  | * |  |
| K09707 | uncharacterized protein | 0.974 | 0.047 | * |  |  | * |
